# A High-Resolution Shape Fitting and Simulation Demonstrated Equatorial Cell Surface Softening during Cytokinesis and Its Promotive Role in Cytokinesis

**DOI:** 10.1371/journal.pone.0031607

**Published:** 2012-02-16

**Authors:** Hiroshi Koyama, Tamiki Umeda, Kazuyuki Nakamura, Tomoyuki Higuchi, Akatsuki Kimura

**Affiliations:** 1 Cell Architecture Laboratory, Center for Frontier Research, National Institute of Genetics, Mishima, Japan; 2 Transdiciplinary Research Integration Center, Research Organization of Information and Systems, Tokyo, Japan; 3 Graduate School of Maritime Sciences, Kobe University, Kobe, Japan; 4 Meiji University, Kawasaki, Japan; 5 The Institute of Statistical Mathematics, Tokyo, Japan; Institute of Science and Technology Austria, Austria

## Abstract

Different models for animal cell cytokinesis posit that the stiffness of the equatorial cortex is either increased or decreased relative to the stiffness of the polar cortex. A recent work has suggested that the critical cytokinesis signaling complex centralspindlin may reduce the stiffness of the equatorial cortex by inactivating the small GTPase Rac. To determine if such a reduction occurs and if it depends on centralspindlin, we devised a method to estimate cortical bending stiffness with high spatio-temporal resolution from *in vivo* cell shapes. Using the early *Caenorhabditis elegans* embryo as a model, we show that the stiffness of the equatorial cell surface is reduced during cytokinesis, whereas the stiffness of the polar cell surface remains stiff. The equatorial reduction of stiffness was compromised in cells with a mutation in the gene encoding the ZEN-4/kinesin-6 subunit of centralspindlin. Theoretical modeling showed that the absence of the equatorial reduction of stiffness could explain the arrest of furrow ingression in the mutant. By contrast, the equatorial reduction of stiffness was sufficient to generate a cleavage furrow even without the constriction force of the contractile ring. In this regime, the contractile ring had a supportive contribution to furrow ingression. We conclude that stiffness is reduced around the equator in a centralspindlin-dependent manner. In addition, computational modeling suggests that proper regulation of stiffness could be sufficient for cleavage furrow ingression.

## Introduction

Cytokinesis is the final step of cell division that mechanically separates a mother cell into two daughter cells. Cytokinesis is accomplished via constriction of a cortical contractile ring. Although the constriction force generated by the actomyosin-based contractile ring is typically considered to be the principal mechanical component for cleavage furrow ingression [1], the mechanical properties of the cell surface also contribute to cleavage furrow ingression [2]. One example that illustrates the importance of cortical mechanics is the fact that furrow ingression is completely inhibited by the disruption of cell surface actin filaments around the polar regions [3]. The relative importance of contractile stress in the ring and modulation of cortical mechanics has not been well characterized. Some gene products required for cytokinesis are involved in cell surface stiffness, e.g., the actin regulator racE of *Dictyostelium discoideum* [4]. For a fundamental understanding of the mechanics of cytokinesis, a consideration of cell surface mechanics is essential.

Cell surface stiffness around the equatorial region has been implied to be locally reduced compared with that around the polar regions. Support for this hypothesis was first provided by the application of drugs targeting the actin cytoskeleton to a restricted region of the cell [3]. Jasplakinolide application to the equatorial region, which stabilizes actin filaments, abolished cleavage furrow ingression, whereas its polar application had no effect on furrow ingression. In contrast, cytochalasin D application around the equatorial region, which disrupts actin filaments, facilitated furrow ingression. Importantly, cytochalasin D application to the polar region abolished furrow ingression. From these results, the “equatorial collapse model” was proposed in which the cell surface should be relatively soft around the equatorial region compared to the polar regions for the furrow to ingress [5]. The molecular mechanism for softening may involve the regulation of the small GTPase Rac [6], the activation of which leads to the formation of actin meshwork structures. A fluorescence resonance energy transfer study in mammalian cells demonstrated that Rac is locally inactivated around the cell equator [7]. A genetic study in *Caenorhabditis elegans* suggested that Rac is inactivated by the conserved cytokinesis regulator centralspindlin, and this regulation is essential for furrow ingression [8]. Centralspindlin is a heterotetrameric complex composed of two molecules of kinesin-6, MKLP1-ZEN-4, and two molecules of MgcRacGAP-CYK-4, which contains a GTPase-activating protein (GAP) domain for Rho family GTPases [6], [9], [10]. One possibility suggested by these data is that centralspindlin promotes cytokinesis by locally reducing cortical stiffness at the cell equator.

To date, there is relatively little experimental information on cortical stiffness during cytokinesis. Measurements with atomic force microscopy (AFM) indicated that the equatorial region was stiffer than the other regions [11]. However, this may not contradict the equatorial softening model because the AFM measurements of equatorial stiffness would likely include the high contractility of the contractile ring in addition to cell surface stiffness. To investigate cell surface stiffness alone, we developed a means to compute cell surface stiffness from *in vivo* cell shapes using a theoretical model based on cortical bending stiffness. Our analysis indicates that the stiffness of the equatorial cell surface is reduced during cytokinesis and that this reduction depends on the centralspindlin component ZEN-4. We also show theoretical predictions for the relative contribution of softening and the contractile ring to furrow ingression.

## Results

### Quantification of cell shape

**Figure 1:**
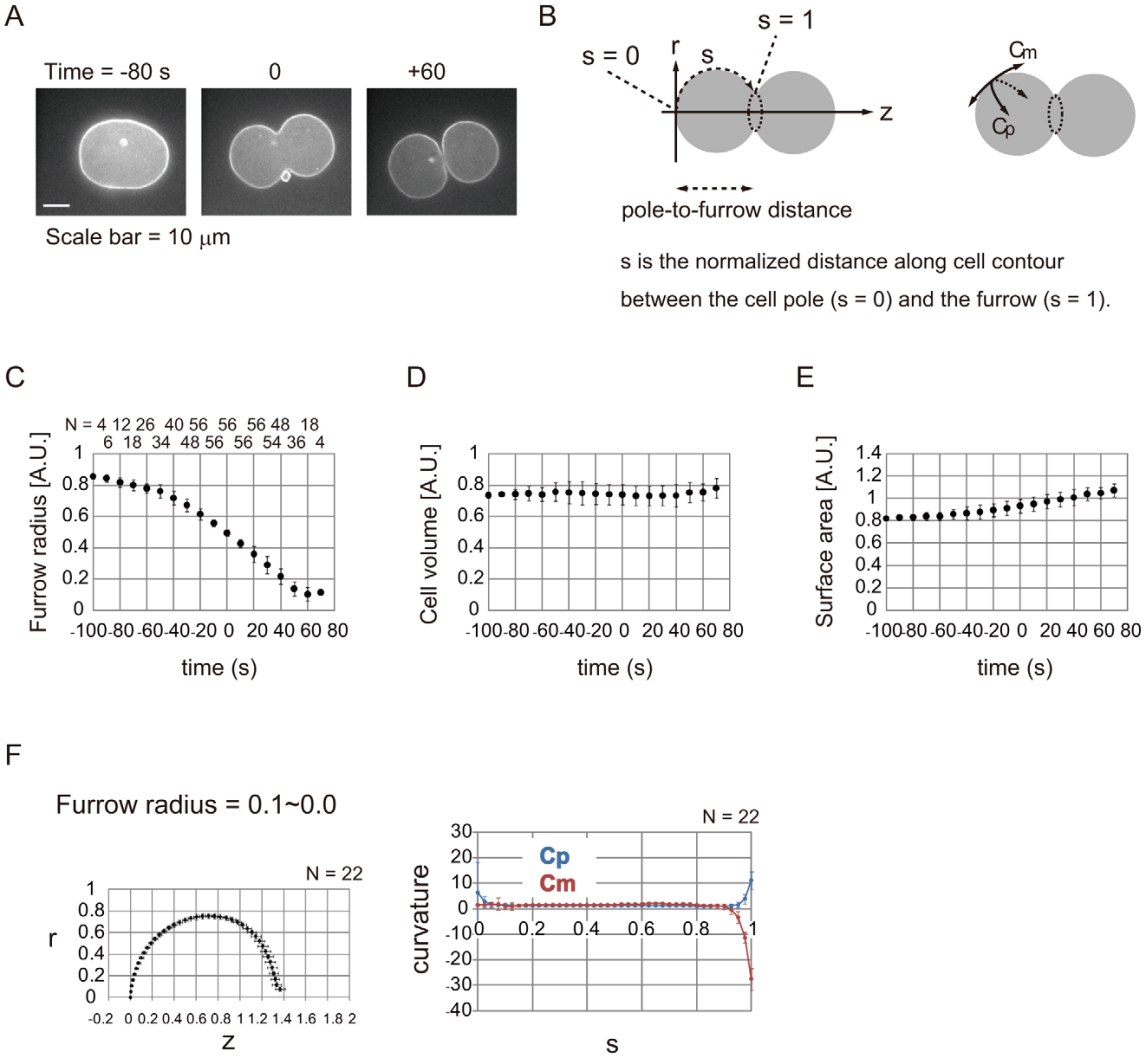
Cell shape quantification. (A) Cytokinesis in an isolated AB cell expressing GFP::PH^PLC1δ1^. Time when furrow radius reached 0.5 unit length, as shown in (C), was defined as 0 s. (B) Definition of coordinates (*r*, *z*, and *s*) and curvatures (*C_m_* and *C_p_*). Cells are assumed to be rotationally symmetrical to the longer axis *z*. The *s* coordinate runs with the cell contour from the cell pole (*s* = 0) to the furrow (*s* = 1). (C, D, E) Time lapse of furrow radius, cell volume, and cell surface area. 1.0 unit furrow radius = 14.5 µm. 1.0 unit volume = 1.27×10^4^ µm^3^. 1.0 unit area = 2.63×10^3^ µm^2^. N for each time point is shown in (C). Error bars denote S.D. (F) Cell shapes (left) and curvatures (right) at the presented furrow radius. *r*, *z*, and *s* are defined in B. 1.0 unit length = 14.5 µm. 1.0 unit curvature is 0.0691 µm^−1^. Error bars denote S.D.

**Figure 2:**
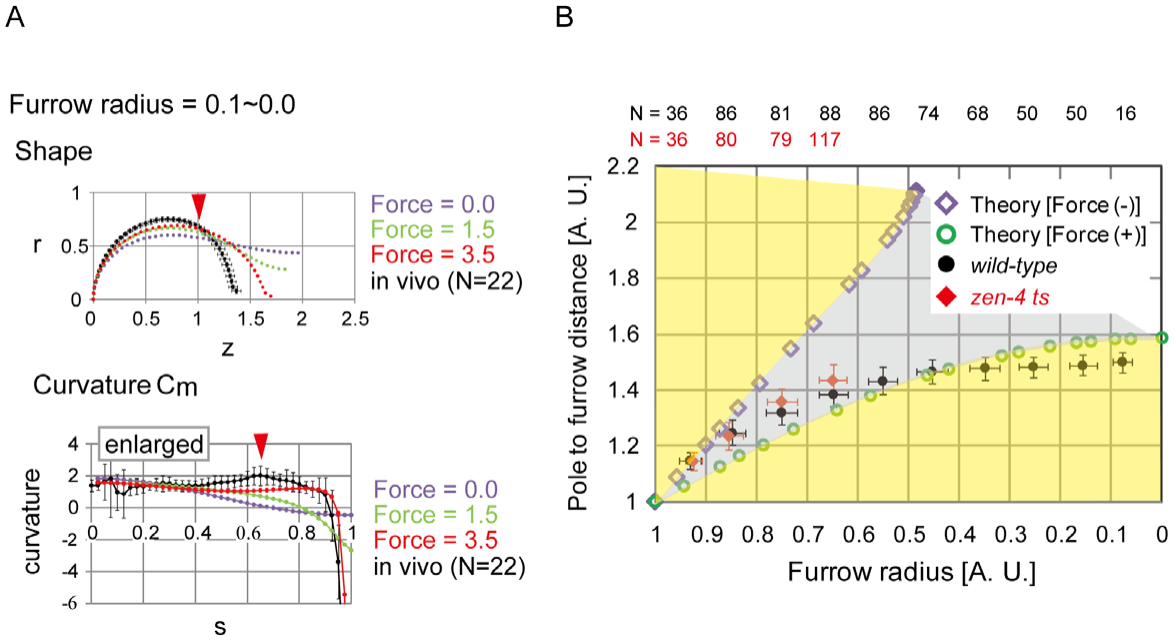
Cell shape under the spatially constant bending modulus. (A) Comparison of cell shapes and curvatures between *in vivo* (black) and the spatially constant *K_c_* model (colored) under the same cell volume and surface area as the *in vivo* cells. The values of the contractile ring force (Force) are shown. Higher *C_m_* was observed *in vivo* (red arrowhead), and the corresponding position is shown in the upper panel. *r*, *z*, and *s* are defined in Fig. 1B. Error bars denote S.D. (B) Comparison of pole-to-furrow distances between the model and *in vivo* (wild-type, black circles and *zen-4* mutant, red diamonds). Pole-to-furrow distances and furrow radii were theoretically calculated in the model with or without the contractile ring force (Force) = ∞ (green open circles or purple open diamonds, respectively). The region surrounded by the green open circles and the purple open diamonds is shown in gray. Regions outside of the gray region are shown in yellow. The distances and radii were normalized by multiplying the length by (cell volume [unit volume])^−1/3^.

To examine whether cell surface stiffness is reduced around the cleavage furrow, we estimated spatio-temporal changes in surface stiffness by fitting *in vivo* cell shapes to a mathematical model. In principle, if we have a mathematical model that allows us to calculate cell shapes under given cell surface stiffness, then conversely, we could predict surface stiffness by using *in vivo* cell shapes. This strategy is similar to that used to predict cell surface tension in sea urchin eggs [12]. To quantify cell shapes, we used *C. elegans* embryonic cells expressing GFP::PH^PLC1δ1^ to label the cell membrane, and we isolated AB cells (Figs. 1A and S1). Their shapes were quantified by a sequence of image processing and cell shape quantification algorithms, including cell contour extraction and curvature calculation (Figs. 1B–F and S1, S2, S3). Throughout this study, we assumed the shapes to be rotationally symmetrical. We defined 1 unit length as 14.5 µm, which corresponds to the radius at the furrow region before ingression. As the furrow ingressed, the cell surface area increased, whereas the cell volume was almost constant (Fig. 1C–E). We used these measured values for shape calculation with our mathematical model. The curvatures along the meridians (*C_m_*) and along the parallels of latitude (*C_p_*) were nearly uniform in the initial phase of cytokinesis (furrow radius = 0.9–0.8) (Fig. S3B). In contrast, *C_m_* was very low around the equatorial region in the final phase (furrow radius = 0.1–0.0) (Fig. 1F), which generates a steep concavity corresponding to the furrow. A noteworthy feature was that *C_m_* was not constant even in the outer region of the furrow, but was slightly larger around the region neighboring the furrow, *s* = 0.6–0.8 (Fig. 2A, red arrowheads, and Fig. S3B, red arrowheads), strongly suggesting that cell surface stiffness is not spatially constant.

### Construction of a mechanical model based on the bending modulus

We then constructed a mathematical model based on the bending elasticity of the cell surface, an index of stiffness. A theory based on bending elasticity has been successfully adopted to explain shape transformations of red blood cells (RBCs) and liposomes [13], [14], [15]. On the basis of this theory, the bending energy of the whole cell surface (*E*) is given as (Text S1- Construction and analyses of the bending model):
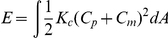
Where, *K*_c_ is the bending modulus, an index of surface stiffness, which was assumed to be spatially constant in RBCs and liposomes, and *A* is the cell surface area. In our cytokinesis model (Text S1- Construction and analyses of the bending model), we assumed that, analogous to RBCs and liposomes, cells transform their shapes while satisfying the global or local minimums of bending energy throughout cytokinesis. Cell volume and surface area were set according to the *in vivo* values (Fig. 1D–E). The major difference from RBCs and liposomes was that *K_c_* was assumed to be spatially inconstant, and *K*_c_ was defined as a function of the cell surface position (*s*) (Fig. 1B). In addition, we introduced a constriction force generated by the contractile ring on the furrow (Text S1- Construction and analyses of the bending model). By providing the spatial distribution of *K*_c_, cell volume and surface area, and the contractile ring force, we could calculate cell shapes with minimums of bending energy.

### Models using a spatially constant bending modulus cannot reproduce *in vivo* cell shapes

Before estimating the spatio-temporal changes in cell surface stiffness by the combination of this model and the *in vivo* cell shapes, we tested whether this model can reproduce the *in vivo* cell shapes for a spatially constant *K_c_*, as various shapes of liposomes can be reproduced under a spatially constant *K_c_* [13], [16]. If the *in vivo* cell shapes were reproduced under this condition, spatially inconstant stiffness is unlikely to be a requirement for cell shape determination. In the absence of the contractile ring force, the calculated shapes were very different from those observed *in vivo* when the same cell volume and surface area were given (Fig. 2A, black vs. purple, and Fig. S4). Although furrow-like shapes were produced by applying the contractile ring force, these shapes were still inconsistent with the *in vivo* shapes (Fig. S4). For example, when we calculated shapes under the same cell volume and surface area as the cell with furrow radius of 0.1–0.0, we could generate a furrow radius of ∼0 by applying a force, but the shape (e.g. the pole-to-furrow distance and the distribution of the curvature) was different (Fig. 2A, green and red). To further assess the differences, we focused on the relationship between the furrow radius and the pole-to-furrow distance (Figs. 1B and 2B). Plots of theoretically calculated shapes with or without the contractile ring force surrounded the gray region (Fig. 2B); therefore, the areas outside of the gray region cannot be reproduced under a spatially constant *K_c_* (Fig. 2B, yellow region). When the furrow radius was smaller than ∼0.4, plots of the *in vivo* cell shapes were located in the yellow region, indicating that the mathematical model cannot reproduce the *in vivo* pole-to-furrow distance even in the presence of the contractile ring force (Fig. 2B). Further analyses revealed that the *in vivo* characteristic feature of the higher *C_m_* around the neighboring region of the furrow was not reproduced in the model (Fig. 2A, *C_m_*, red arrowhead). Therefore, when a spatially constant *K_c_* is assumed, *in vivo* cell shapes with deeper furrows are not reproduced by the bending model.

### Cell shape analysis suggests that cortical stiffness is reduced at the cell equator as cytokinesis proceeds

**Figure 3:**
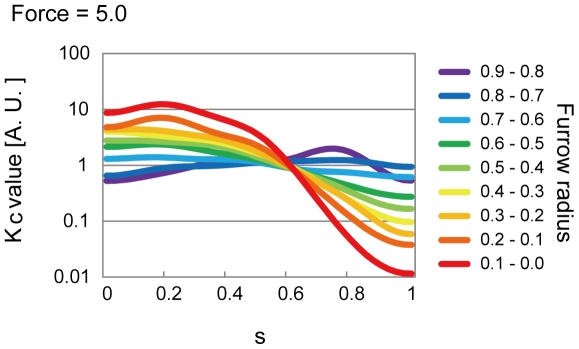
Spatio-temporal changes in cortical stiffness. Estimated spatio-temporal changes in cortical stiffness *K_c_* is shown. Cell shape analysis indicates that cortical stiffness is dramatically reduced at the cell equator as cytokinesis proceeds. The estimation was performed for a contractile ring force (Force) = 5.0 or other values (Figs. 5A and S8). *s* is defined in Fig. 1B.

We then searched for a spatial distribution of *K_c_* that could reproduce the *in vivo* cell shapes. Since we could not analytically calculate the spatial distribution of *K_c_*, we constructed an optimization algorithm. In this algorithm, we first arbitrarily produced a spatial distribution of *K_c_*, and calculated the shape in the bending model. The spatial distribution of *K_c_* was then repeatedly improved to minimize the difference between the *in vivo* and theoretically calculated shapes (Text S1- Estimation of the spatio-temporal changes in the bending modulus). Spatial distributions of *K_c_* that generated shapes in good agreement with the *in vivo* shapes were successfully estimated (Figs. 3, S5, S6, S7, S8). In the initial phase of cytokinesis, *K_c_* values were spatially almost constant (Fig. 3, purple). In the final phase, *K_c_* values around the furrow were lower than those around the polar regions (Fig. 3, red). These overall patterns were conserved even under any values of the contractile ring force (Fig. 3, Force = 5.0, and Fig. S8, Force = 20.0 and 50.0). These results suggest that cell surface stiffness is dynamically controlled during cytokinesis. Our results indicate that cortical stiffness is dramatically reduced at the cell equator concurrent with furrow ingression during cytokinesis.

### Modeling predicts that reduction of cortical stiffness at the cell equator during cytokinesis is impaired in *zen-4* mutant cells

**Figure 4:**
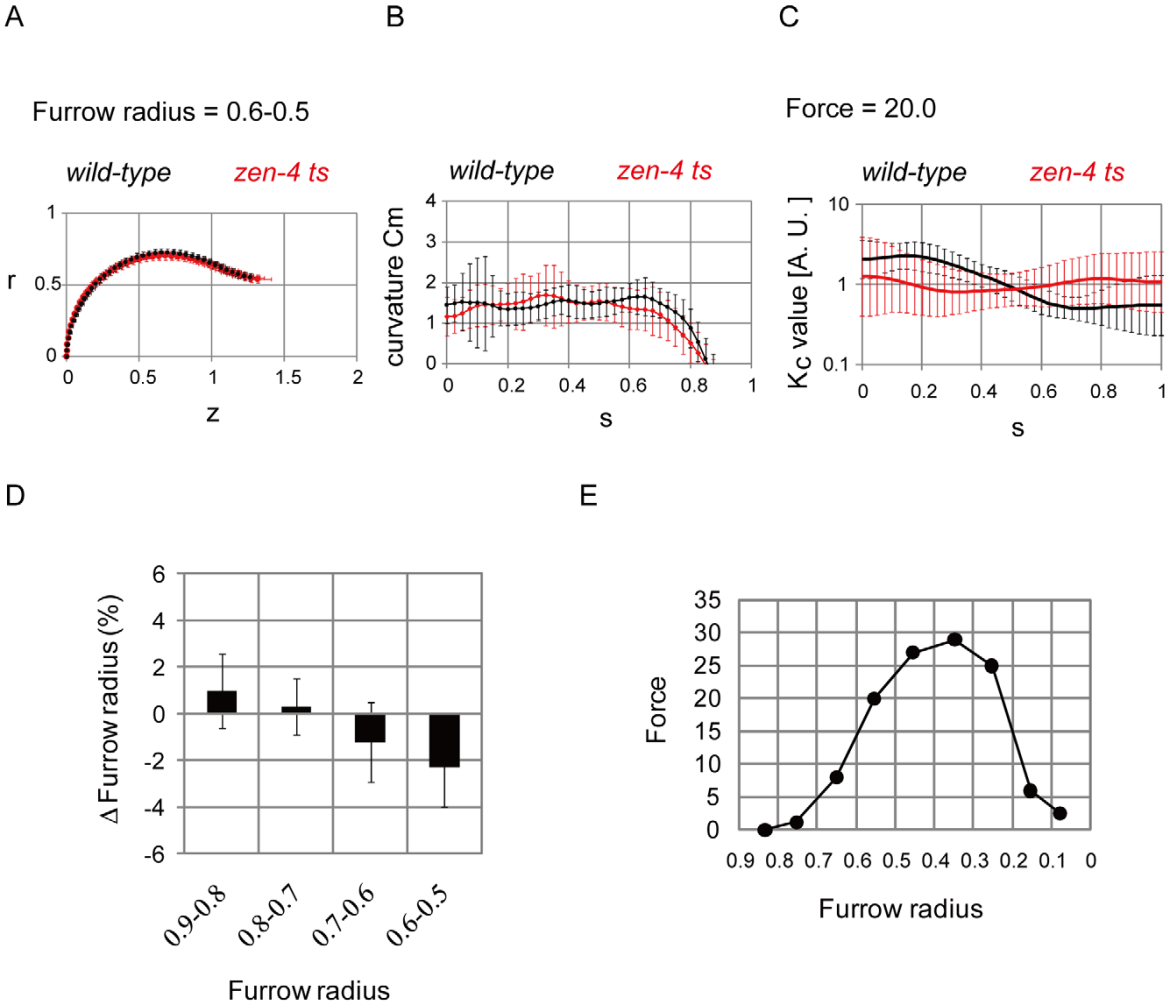
Analyses in *zen-4* mutant cells. (A, B, C) Cell shapes (A), curvature *C_m_* (B), and estimated cortical stiffness *K_c_* for a contractile ring force (Force) = 20.0 (C) at a furrow radius = 0.6–0.5 are shown with S.D. for the wild-type (N = 94 in A and B, N = 30 in C) and mutant cells (N = 126 in A and B, N = 117 in C). P-value<0.05 at *s* = 0.7 in (B) and *s* = 1.0 in (C). *r*, *z*, and *s* are defined in Fig. 1B. (D) Changes in the mutant cells' furrow radius by introducing *K_c_* derived from the wild-type cells were calculated for each furrow radius = 0.9–0.8∼0.6–0.5. N = 26, 37, 27, and 30, respectively. (E) The magnitudes of the contractile ring force (Force) required to achieve the presented furrow radii were calculated for a spatially constant *K_c_* ( = 1.0).

What is the molecular regulator of the equatorial reduction in cell surface stiffness? Centralspindlin is a molecular complex essential for furrow ingression: in the *C. elegans* embryo, mutation or depletion of centralspindlin components causes the arrest of furrow ingression at approximately the half-way point of closure [8]. Centralspindlin is a key cytokinesis regulator that has been shown to localize to the spindle midzone as well as to the equatorial cortex [6], [9]. Centralspindlin contains a GAP activity that is essential for its role in furrow ingression [8]. The centralspindlin GAP has been proposed to inhibit the small GTPase Rac, which activates the ARP-2/3 complex to promote the formation of branched actin networks [8]. Consequently, one attractive hypothesis is that centralspindlin reduces cortical stiffness at the cell equator by inactivating Rac [6], [8]. We focused on ZEN-4/Kinesin-6, a component of centralspindlin, and examined whether the dramatic equatorial reduction of cell surface stiffness was diminished in *zen-4* mutant cells. First, we quantified the shapes of *zen-4* mutant cells. At a furrow radius of 0.6–0.5, where the mutant cells arrested furrow ingression, there was a difference between the cell contours of the mutant and wild-type cells (Fig. 4A). In particular, the pole-to-furrow distances in the mutant cells were larger than in the wild-type cells (Figs. 2B and 4A). For *s* = 0.6–0.8, where *C_m_* was increased in the wild-type cells in the later phases (Figs. 2A and S3B), this increase disappeared in the mutant cells (Figs. 4B, S3C–D, and S10). The difference in curvature between the wild-type and *zen-4* mutant cells was statistically significant, for example at *s* = 0.7 (*P*<0.05, Fig. 4B). We then estimated *K_c_* under various contractile ring forces (Figs. 4C and S9). *K_c_* was almost spatially constant in the mutant cells under a constriction force = 20.0 and a furrow radius = 0.6–0.5 (Fig. 4C). *K_c_* around the furrow or around the polar region was higher or lower in the mutant cells than in the wild-type cells, respectively (Figs. 4C and S9, furrow radius = 0.6–0.5). These results strongly suggested that ZEN-4 is responsible for the reduction of cell surface stiffness around the furrow.

### Failure in the reduction of cell surface stiffness around the cleavage furrow in *zen-4* mutant cells impaired furrow ingression

The *zen-4* mutant cells failed to reduce cell surface stiffness around the furrow (Fig. 4C) and displayed furrow ingression arrest [17]. We hypothesized that the failure to reduce cell surface stiffness caused the arrest of furrow ingression in these cells; therefore, the forced reduction of stiffness around the furrow in these cells would suppress this arrest. Theoretically, if we reduced stiffness around the furrow in these cells in our bending model, deeper furrows should be formed. The *K_c_* values around the furrow in the mutant cells were forcedly reduced by introducing the *K_c_* derived from the wild-type cells at a furrow radius of 0.6–0.5 (Fig. 4C), and we then calculated the shapes and their furrow radii. This introduction yielded ∼2% deeper furrows than the original furrows (Fig. 4D, furrow radius = 0.6–0.5). By contrast, in the mutant cells at a furrow radius = 0.9–0.8∼0.7–0.6, the introduction of *K_c_* derived from the wild-type cells at the corresponding furrow radius (Fig. S9, furrow radius = 0.9–0.8∼0.7–0.6 with Force = 20.0), showed no or a weaker effect on the formation of deeper furrows (Fig. 4D, furrow radius = 0.9–0.8∼0.7–0.6). These results supported the idea that the failure to reduce stiffness in the mutant cells blocks furrow ingression. A similar argument is possible without calculating the shapes, but from the larger surface area to cell volume ratio in the mutant cells than in the wild-type cells (Fig. S11).

We further investigated the effect of the defective regulation of stiffness on furrow ingression. Under a spatially constant *K_c_*, we calculated the magnitude of the contractile ring force required for the furrow to reach a given radius (Fig. 4E). The required force increased dramatically with a radius <0.6, indicating the increased resistive effect of the cell surface against furrow ingression. Intriguingly, this timing was consistent with the timing of the arrest of furrow ingression in the mutant cells. In the mutant cells, we predict that the force required to constrict the furrow less than a radius of ∼0.6 becomes larger than the force that the contractile ring can produce because of the defect in regulating cell surface stiffness, leading to furrow ingression arrest.

#### Equatorial reduction of cell surface stiffness promoted furrow ingression

**Figure 5:**
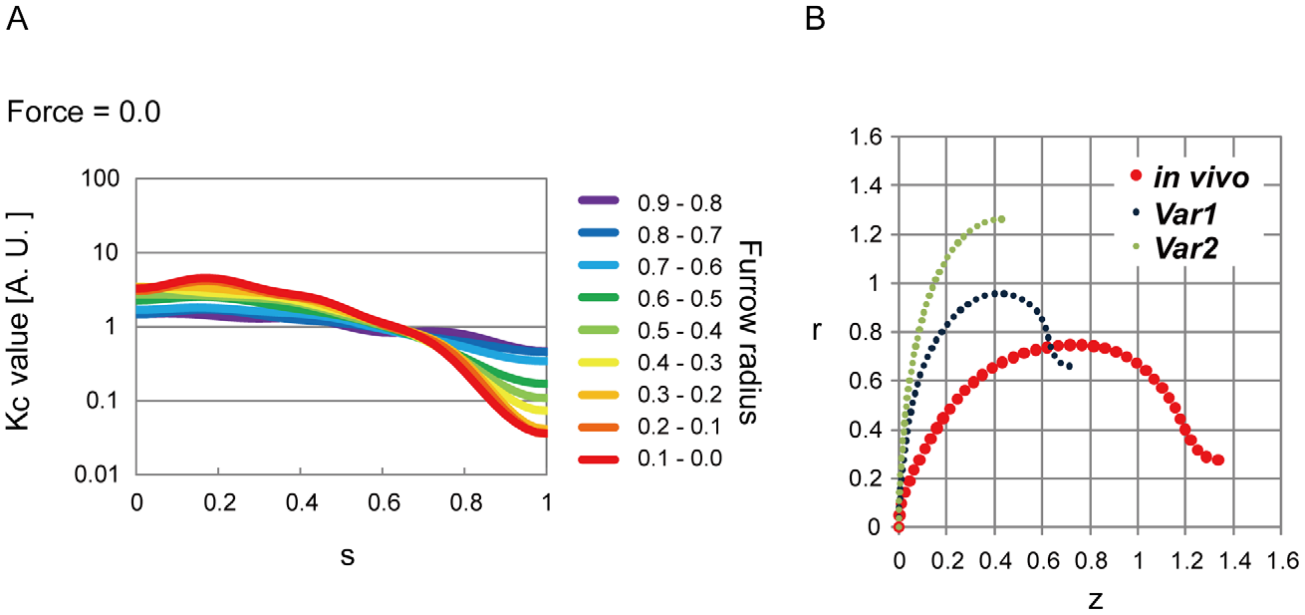
Promoting effect of cell surface stiffness on furrow ingression. (A) Spatio-temporal changes in cortical stiffness *K_c_* estimated in the absence of the contractile ring force (Force). (B) Calculated shapes under the same estimated spatial distribution of *K_c_* at furrow-radius = 0.3–0.2 in (A). “*in vivo*” coincides with the *in vivo* cell shape, while “Var1-2” do not. Bending energy for each shape is shown in Figure S12. *r*, *z*, and *s* are defined in Fig. 1B.

Figure 4D suggested that the reduction of cell surface stiffness around the furrow can decrease the magnitude of the contractile ring force required for furrow ingression. Further analyses showed that the magnitude of the contractile ring force could be ultimately 0; even in the absence of the contractile ring force, we found spatial distributions of *K_c_* that reproduced the *in vivo* cell shapes (Figs. 5A and S7). The estimated overall patterns of *K_c_*, which included the reduction of stiffness around the furrow, were similar to those in the presence of the contractile ring force (Fig. 3 vs. 5A). Importantly, the absence of the contractile ring force did not demand a further increase in the reduction of stiffness around the furrow than in the presence of the contractile ring force (Figs. 3 and 5A, furrow radius = 0.6–0.5∼0.1–0.0). These observations indicated that the proper regulation of *K_c_* could promote furrow ingression, and further raised the possibility that the regulation of *K_c_* was sufficient for furrow ingression. A previous study proposed a similar hypothesis in which the reduction of stiffness mediated by Rac around the furrow may be sufficient to complete cytokinesis in some mammalian cell types [6], [18].

If the *in vivo* cell shapes were really most energetically favorable in the absence of the contractile ring force, the regulation of *K_c_* was sufficient for furrow ingression. We examined whether there were other shapes that were more energetically favorable than the *in vivo* shapes. Under the spatial distribution of *K_c_* estimated in the absence of the contractile ring force, there were at least 2 extra shapes (Var1–2) with a bending energy minimum other than the shape consistent with the *in vivo* shape (*in vivo*), which also had a minimum (Fig. 5B). Var1 and 2 in Figure 5B had lower bending energy compared with the *in vivo* shape (Fig. S12A), suggesting that the *in vivo* shape was not most favorable, but tended to expand its furrow radius toward Var1 and Var2. The *in vivo* shape would be stabilized by the contractile ring by preventing furrow expansion (Fig. S12B-C). Thus, it is possible that furrow ingression is driven by the regulation of *K_c_* with shape stabilization by the contractile ring (Fig. S12C).

## Discussion

**Figure 6:**
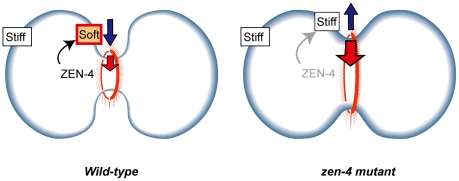
Model of regulation of cell surface stiffness and its contribution to furrow ingression. In the absence of ZEN-4, the cell surface generates a resistive force against furrow ingression (right panel; blue arrow). In the presence of ZEN-4, cell surface stiffness around the furrow is locally reduced, which decreases the resistive force and promotes furrow ingression (left panel; blue arrow). Thus, the weaker contractile ring force (red arrow) becomes sufficient for furrow ingression.

In the present study, we constructed a method to estimate spatio-temporal changes of cell surface stiffness (*K_c_*) by using *in vivo* cell shapes with a bending model. This approach provided the first direct evidence for the hypothesis [3], [7] that cell surface stiffness around the furrow is locally reduced compared to the polar regions (Figs. 3 and 6). Furthermore, the reduction of stiffness is regulated by ZEN-4 (Figs. 4C and 6). Thus, we support the hypothesis that local inactivation of Rac by the ZEN-4-containing centralspindlin complex around the furrow leads to the disruption of actin meshwork structures, leading to the reduction of stiffness.

We theoretically analyzed the contribution of the equatorial reduction of stiffness to furrow ingression. The relative reduction of stiffness can decrease the magnitude of the required constriction force of the contractile ring for furrow ingression (Fig. 4D–E), and ultimately, the magnitude can reach 0 under optimized spatial distributions of stiffness (Fig. 5A). These results theoretically demonstrate that the reduction can qualitatively change the contribution of the cell surface to furrow ingression from resistive to promotive (Fig. 6). The promotive function of the cell surface should be significant for furrow ingression *in vivo* because the defect in this function appears just before the arrest of furrow ingression in the *zen-4* mutant cells (Fig. 4D). It has been reported that cytokinesis can be accomplished without the constriction of contractile ring in some animal cell types [19]. The promotive function of the cell surface may drive such contractile ring-independent cytokinesis (Fig. S12).

In addition to bending elasticity, contractility/surface tension can be a target of local control in cell surface mechanics [12]. An estimation of the cell surface's total energy provided from surface tension and bending elasticity in animal or amoeba cells argues that the former has a greater contribution to cell shape determination [19], [20]. A popular model for surface tension is the polar relaxation model [21], where relaxation of surface tension around the polar regions facilitates the formation and constriction of the contractile ring. Adjustment of surface tension throughout the surface can reproduce cell shapes in sea urchin eggs [12]. However, surface tension models cannot explain why the local application of cytochalasin D or a myosin inhibitor, blebbistatin, around the polar regions inhibited furrow ingression [3], [22]; their application should facilitate the constriction of the contractile ring in the context of the polar relaxation model because it induces the polar relaxation of surface tension. Thus, we suppose that surface tension has a partial or slight effect on the determination of cell shape transformation, at least in some cell types. Conversely, our model based on *K_c_* can explain the inhibition by the drugs because the increased stiffness around the polar regions can promote furrow ingression. Moreover, we found no or only slight defects in the spatial distribution of surface tension just before the arrest of furrow ingression in the *zen-4* mutant cells, as far as we estimated surface tension on the basis of a surface tension model (Figs. S13 and S14). Hence, it seems unlikely that the arrest of furrow ingression in the mutant cells arises from defects in surface tension control, although we cannot rule out the possibility that surface tension significantly contributes to furrow ingression, especially in phases other than the middle phase where the mutant cells are arrested. Similarly, it is possible that components other than *K_c_* or surface tension may affect furrow ingression, e.g., astral microtubules and mechanical features of the cytoplasm [5], [23]. Although we do not know all of the mechanical components for cytokinesis [1], our simplified model based on *K_c_* successfully explained the *in vivo* phenomena, e.g., the arrest in the mutant cells and the consequences of the local application of drugs.

One of the advantages of our estimating method is that it allowed us to estimate *K_c_* with a high spatio-temporal resolution, which has not been separable from contractility/surface tension in AFM [11]. Actin-based cytoskeletal dynamics could be predicted through the estimation of *K_c_*. In addition, our method only requires imaged cell shapes, and thus, it is easily applicable to microscopic images of cell shapes. Conversely, it should be noted that this method may be sensitive to several factors such as the spatial resolution of measurements of *in vivo* cell shapes and the smoothness cost for spatial changes in *K_c_* (Text S1- Estimation of the spatio-temporal changes in the bending modulus). We did not detect any stiffer regions coinciding with the contractile ring (Fig. 3), which may be due to an insufficiency in the spatial resolution of the furrow and/or the smoothness cost, which disfavors acute spatial changes in *K_c_*.

According to our estimation, *K_c_* can spatially vary by ∼2 orders (Figs. 5 and S7). Although there are no experimental data for *K_c_* with a spatial resolution, Young's modulus of cells measured by AFM spatially varied by ∼2 orders [24], suggesting that cell surface stiffness can spatially change by this magnitude.

The mechanics of cytokinesis remain enigmatic. Although many direct and indirect regulators of the cytoskeleton are involved in cytokinesis, their mechanical actions are poorly understood [4]. The mechanical actions of several proteins are theoretically related to experimentally observed cytokinesis dynamics [20]. Our method to estimate cell surface stiffness can be useful to predict the mechanical actions of proteins in a spatio-temporal manner.

## Materials and Methods

### Isolation of AB cells

The eggshell and vitelline membrane of *C. elegans* embryos were removed as described previously [25], [26] with some modifications (Text S1- Materials and manipulation of *Caenorhabditis elegans* cells). Since the chitinase/α-chymotrypsin digestion was performed at ∼26°C which is the restriction temperature for the *zen-4* mutant cells, the *zen-4* mutant embryos were digested before 1-cell stage cytokinesis occurred, and then moved to the permissive temperature for cytokinesis to proceed. After that, the vitelline membrane was removed using a glass capillary.

#### Image processing and quantification of cell shape

Image processing and cell shape quantification algorithms were written in C. In brief, image binarization was performed to extract cell contour, cell contour was traced, and the values of *r-z* coordinate and curvatures of cell contour were calculated (Text S1- Image processing). Importantly, we assumed the shape to be rotationally symmetrical to the longer axis and to be symmetrical to the cell equator. Thus, we separated a cell into 4 quadrants by its rotational axis and the cell equator (Text S1- Quantification of cell shape).

### Construction of the bending model

If cells were assumed to transform their shapes while satisfying minimums of bending energy under constraints of cell volume and surface area, the conditions that cell shapes should satisfy could be derived from the variation of *δH* = 0.
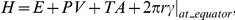
where *E* is the bending energy, *V* is the cell volume, *A* is the cell surface area, *r* at equator is the radius of the equator, and *P*, *T*, and γ are Lagrange multipliers (Text S1- Construction and analyses of the bending model). γ can be interpreted as the line tension/constriction force generated by the contractile ring.

### Estimation of the bending modulus

The optimization algorithm for the estimation of the bending modulus was shown in brief. An initial spatial distribution of *K_c_* was arbitrarily provided. The value of the contractile ring force was also given. A shape with minimum bending energy was then calculated. The difference between the calculated and *in vivo* shapes was evaluated by defining a cost function (Text S1- Estimation of the spatio-temporal changes in the bending modulus). Then, the spatial distribution of *K_c_* was improved to decrease the value of the cost function with the quasi-Newton method.

Other theoretical analyses and the *C. elegans* strains used in this study were described in Text S1.

## Supporting Information

Text S1 **The details of materials and manipulation of** ***Caenorhabditis elegans*** **cells, image processing, quantification of cell shape, construction and analyses of the bending model, estimation of the spatio-temporal changes in the bending modulus, and surface tension model.**
(PDF)

Figure S1 **Microscopy images of isolated AB cells.** (A) AB cells were isolated from 2-cell stage embryos as described in Section 1 (Materials and manipulation of *Caenorhabditis elegans* cells). (B) Time-lapse images of a wild-type AB cell expressing GFP::PH^PLC1δ1^. Scale bar, 10 µm. (C) Time-lapse images of a *zen-4 ts* mutant AB cell expressing GFP::PH^PLC1δ1^. Scale bar, 10 µm.
(PDF)

Figure S2 **Image processing and quantification of cell shapes.** (A) Overview of the procedures. The first panel is a raw microscopy image. The second panel is after the extraction of the CCPs (Cell Contour Pixels). The third panel is the determination of the rotational axis (dashed line). The final panel is the separation of a cell into 4 quadrants (q1–q4) by the rotational axis (horizontal dashed line) and equatorial plane (vertical dashed line). Cell shape parameters, including the values of the *r-z* coordinates, curvatures, and cell volume and surface area, were independently quantified for each quadrant. (B) Image binarization by local thresholding. The positions of pixel **a** and pixels **b_1–2_**–**e_1–2_** are shown. The intensity of **a** was compared with that of **b_1–2_**–**e_1–2_**. See Text S1 for details. (C) Selection of BP1s (Boundary Pixels). CCPs are shown in light and dark gray. BP1s were selected from CCPs along the boundary between CCPs and the cytoplasmic region by a boundary-following algorithm (dark gray). (D) Selection of BP2s. (Left panel) The positional relationship of BP2s (i−1, i, and i+1) is shown. BP2s were selected from BP1s that were located along the boundary between CCPs (gray) and the cytoplasmic region. i+1 was selected so that the distance between Line1 (L1) and i+1 was larger than 2 pixels. (Right panel) In the case for an acutely curved region, where the angle between Line1 (L1) and Line2 (L2) was smaller than π/2 degrees, an additional pixel, h, was inserted. See Text S1 for details. (E) Determination of normal vectors. A circle that runs through i−1, i, and i+1 is shown (dashed circle). The normal vector for i was defined as the solid line that ran through i and the center (diamond) of the circle. (F) Calculation of the curvature *C_m_*. The crossing point (diamond) and the angle *dθ* between the normal vectors for i and for i+1 are shown. The curvature *C_m_* of the arc sandwiched between i and i+1 was defined as the reciprocal of the distance between the crossing point and the arc.
(PDF)

Figure S3 **Quantified values of the** ***r–z*** **coordinates and curvatures.** (A) The values of the *r–z* coordinate of the wild-type (black) and *zen-4 ts* (red) cells are shown for each furrow radius. The majority of the *zen-4 ts* cells arrested the furrow at a furrow radius of 0.6–0.5; thus, the values of the *r–z* coordinates in the *zen-4 ts* cells for a furrow radius <0.5 are not shown. N = 48, 70 (0.9–0.8), 96, 81 (0.8–0.7), 86, 96 (0.7–0.6), 94, 126 (0.6–0.5), 98, not shown (n.s.) (0.5–0.4), 76, n.s. (0.4–0.3), 62, n.s. (0.3–0.2), 53, n.s. (0.2–0.1), and 22, n.s. (0.1–0.0) for each furrow radius (in parentheses) in the wild-type or *zen-4 ts* cells, respectively. (B and C) The curvatures *C_m_* (red) and *C_p_* (blue) in the wild-type cells (B) or *zen-4 ts* cells (C) are shown for each furrow radius. The right panels are enlarged from the left ones. A region with a higher *C_m_* in the wild-type cells is shown (B, red arrow heads). The larger error bars of *C_p_* at *s*<0.2 may be caused by measurement errors, as described in Section 3 Quantification of cell shape. N for each furrow radius in the wild-type or *zen-4 ts* cells is shown in A. (D) Comparison of *C_m_* between the wild-type and *zen-4 ts* cells. *C_m_* in the wild-type cells from (B) and in the *zen-4 ts* cells from (C) are presented.
(PDF)

Figure S4 **Bending model with spatially constant** ***K_c_*** **.** (A) Definition of coordinates. The *t* coordinate starts from a cell pole (*t* = 0), runs along the cell contour, and ends at the furrow (*t* = *t_1_*). Normalization of the *t* coordinate by *t_1_* generates the *s* coordinate. *θ* is the angle between the rotational axis *z* and the normal vector of the cell contour. (B) Schematic illustration of the bending model with spatially constant *K_c_*. “Force” indicates the constriction force of the contractile ring. (C) Relationships between the contractile ring forces and furrow radii and between the contractile ring forces and pole-to-furrow distances. In the calculation of shapes, cell volume and surface area were fixed. Both parameters were derived from the average values in the wild-type cells at the presented furrow radii (0.0–0.1, 0.1–0.2, 0.4–0.5, and 0.8–0.9). (D) Comparison of shapes between the wild-type cells and the bending model. The shapes and curvature *C_m_* in the wild-type cells (N = 53) and those obtained in (C) (0.2–0.1) are shown for each value of the force. See the Text S1 for a description of the calculation for the shape under a force = ∞.
(PDF)

Figure S5 **Estimation of the spatio-temporal changes in** ***K_c_*** **by Method 2 using cosine curves.** *K_c_* values are shown in a non-logarithmic (left panel) or logarithmic manner (right panel).
(PDF)

Figure S6 **Effect of the weight of the smoothness cost on estimating the spatial changes in** ***K_c_*** **by Method 3.** (A) Two examples of *in vivo* cell shapes are shown. (B) Estimated spatial patterns of *K_c_* for the 2 *in vivo* cell shapes are shown under the different weight (*ω_1_*) of the smoothness cost.
(PDF)

Figure S7 **Shapes under the estimated spatial patterns of** ***K_c_*** **.** (A) Schematic illustration of the bending model with spatially inconstant *K_c_*. “Force” indicates the constriction force of the contractile ring. (B) Examples of estimated *K_c_* values in the presence or absence of contractile ring force. (C) Comparison of shapes calculated under the *K_c_* values shown in (B) with the *in vivo* shapes. The shapes in the model were in good agreement with the *in vivo* cell shapes. (D) Comparison of the curvature *C_m_* calculated under the *K_c_* values shown in (B) with the *in vivo* curvature. The higher *C_m_* region around the neighboring regions of the furrow was accurately reproduced under these *K_c_* values, suggesting that the higher *C_m_* was linked to the spatially inconstant distribution of *K_c_*.
(PDF)

Figure S8 **Estimation of the spatio-temporal changes in** ***K_c_*** **by Method 3.** *K_c_* values estimated under Force = 20.0 (left panel) and  = 50.0 (right panel) are shown.
(PDF)

Figure S9 **Comparison of the spatio-temporal changes in** ***K_c_*** **between wild-type and** ***zen-4 ts*** **cells.** (A) *K_c_* estimated under Force = 20.0 or  = 0.0 for the presented furrow radii. Black, wild-type cells; red, *zen-4 ts* cells. (B) *K_c_* estimated under Force = 50.0 in the wild-type cells are shown. The *K_c_* values were still spatially inconstant.
(PDF)

Figure S10 **Comparison of the** ***in vivo*** **cell shapes in wild-type and** ***zen-4 ts*** **cells with those calculated under a spatially constant** ***K_c_*** **.** (A) Cell shapes were calculated in the bending model with a spatially constant *K_c_* under Force = 20. The calculated shapes and the *in vivo* cell shapes in the wild-type and *zen-4 ts* cells were compared for a furrow radius = 0.6–0.5. N = 94 (wild-type) and 126 (*zen-4 ts*). (B) The curvatures *C_m_* of the cell shapes in (A) are shown. The higher *C_m_* around *s* = 0.6–0.8 observed in the wild-type cells disappeared in the *zen-4 ts* cells, and *C_m_* in the *zen-4 ts* cells was consistent with that in the bending model under a spatially constant *K_c_*. N = 94 (wild-type) and 126 (*zen-4 ts*).
(PDF)

Figure S11 **Comparison of the** ***in vivo*** **cell surface area in wild-type and** ***zen-4 ts*** **cells.** The cell surface area normalized by cell volume was calculated for each furrow radius. The *zen-4 ts* cells had a slightly larger surface area than the wild-type cells. As a larger surface area is generally advantageous to form a deeper furrow, these results indicate that the status of the mechanical property, rather than the surface area, was more resistant to furrow ingression in the mutant cells than in the wild-type cells. N = 48, 70 (0.9–0.8), 96, 81 (0.8–0.7), 86, 96 (0.7–0.6), 94, 126 (0.6–0.5), and 98, n.e. (0.5–0.4) for each furrow radius (in parentheses) in the wild-type or *zen-4 ts* cells, respectively.
(PDF)

Figure S12 **Comparison of the bending energy among various shapes with the same** ***K_c_*** **values.** (A) The values for the bending energy of various shapes presented in Figure 5B were calculated for each time point. Note that Figure 5B corresponds to a time of 40 s. These shapes had the same spatial distributions of *K_c_* and were calculated under Force = 0, as described in Figure 5B. (B) The furrow radius and pole-to-furrow distance of each shape are shown. (C) Schematic illustration of the energy landscape and possible roles of bending elasticity, the contractile ring, and the spindle. An energy landscape at one time point was expected from (A) and (B). Note that “*in vivo*,” Var1, and Var2 were located at the global or local minimums of the bending energy (data not shown). Var1 and Var2 have larger furrow radii (→), smaller pole-to-furrow distances (←), and lower bending energy (black) than the *in vivo* shape. Therefore, additional energy sources (blue) may be required to stabilize the *in vivo* shape and avoid shape transformation from the *in vivo* shape to Var1 or Var2. One candidate was line tension energy derived from the contractile ring, which corresponds to *2πrγ* in equation 4 in Section 4-1. However, the line tension energy was 0 in this analysis because contractile ring force (*γ*) was assumed to be absent. Nevertheless, it may be possible that the contractile ring can provide the energy source through another mechanism such as a kind of ratchet that confers a large amount of energy against furrow expansion, but no energy for furrow ingression. Spindle or cell adhesion, if it is present, may also provide an energy source. The sum of the energy landscape (black+blue) is shown in red, in which the *in vivo* shape is stabilized. Consistent with this ides, cell adhesion is critical for the progression of the contractile ring-independent cytokinesis in *Dictyostelium discoideum* and mammalian cultured cells [19], [27], [28]. The contractile ring-independent cytokinesis might be driven by the combination of cell surface stiffness and cell adhesion.
(PDF)

Figure S13 **Estimation of the spatio-temporal changes in surface tension in the spatially inconstant surface tension model.** (A) Schematic illustration of the surface tension model. (B) The estimated surface tension *T_p_* and *T_m_* are shown. The high *T_p_* region around the furrow would correspond to the contractility of the contractile ring.
(PDF)

Figure S14 **Comparison of the estimated spatio-temporal changes in surface tension** ***T_p_*** **between wild-type and** ***zen-4 ts*** **cells.** The values of the estimated surface tension *T_p_* for each furrow radius are shown for wild-type and *zen-4 ts* cells.
(PDF)
