# Supplementary material for: A High-Resolution Shape Fitting and Simulation Demonstrated Equatorial Cell Surface Softening during Cytokinesis and Its Promotive Role in Cytokinesis
Source: PLoS One. 2012 Feb 16;7(2):e31607. doi: 10.1371/journal.pone.0031607 (PMC3281004; doi:10.1371/journal.pone.0031607)
Supplement: Text S1 — The details of materials and manipulation of Caenorhabditis elegans cells, image processing, quantification of cell shape, construction and analyses of the bending model, estimation of the spatio-temporal changes in the bending modulus, and surface tension model. (PDF) [file pone.0031607.s001.pdf]

Supporting Text S1

Contents

1. Materials and manipulation of *Caenorhabditis elegans* cells
2. Image processing
3. Quantification of cell shape
4. Construction and analyses of the bending model
5. Estimation of the spatio-temporal changes in the bending modulus
6. Surface tension model
7. References in supporting information

1. Materials and manipulation of *Caenorhabditis elegans* cells

Strains

C. elegans strains were maintained with the standard techniques [1]. The following strains were used in this study: *C. elegans* embryonic cells expressing GFP::PH$^{PLC1\delta1}$ (OD58) to label the cell membrane [2] and *zen-4* temperature sensitive (*zen-4 ts*) cells expressing GFP::PH$^{PLC1\delta1}$ (CAL0252) obtained by mating OD58 and EU716 (*zen-4(or153) IV*) [3]. OD58 was kindly provided from K. Oegema (University of California, San Diego) and EU716 was from the *Caenorhabditis* Genetics Center, funded by the National Institutes of Health.

AB cell isolation

In the present study, we used isolated AB cells whose eggshell and vitelline membrane surrounding *C. elegans* embryos were removed to avoid mechanical constraints from these structures and cell adhesion (Fig. S1). Removal of the eggshell and vitelline membrane was performed as described previously [4,5] with the following modifications. In brief, embryos were treated with ~0.25% hypochlorite (Nacalai Tesque, Kyoto, Japan) for 1.5–2.5 min, and digested with chitinase (chitinase from *Streptomyces griseus*; Sigma-Aldrich, St. Louis, Mo, USA)/α-chymotrypsin (Sigma-Aldrich) for 3.5–8.0 min. Chitinase/α-chymotrypsin were dissolved in Egg HEPES buffer (118 mM NaCl, 48 mM KCl, 25 mM HEPES pH 7.2). The chitinase/α-chymotrypsin digestion was performed at ~26°C. Since 26°C is the restriction temperature for the *zen-4 ts* cells, *zen-4 ts* embryos were digested before 1-cell stage cytokinesis occurred, and then moved to the permissive temperature for cytokinesis to proceed. The remaining vitelline membrane was then removed by drawing the embryos in and out of a glass capillary. Finally, AB and P1 cells were separated with a glass capillary (Fig. S1A). Culture medium was used as described previously [5].

Microscopy

The isolated AB cells were imaged at ~26°C. GFP fusion proteins were visualized with a

spinning-disk confocal system (CSU10; Yokogawa, Tokyo, Japan) mounted on a microscope (BX61; Olympus, Tokyo, Japan) equipped with a UPlanSApo 100× 1.40 NA objective (Olympus). Digital images were obtained with a CCD camera (iXon; Andor, Belfast, UK) controlled by IPLab software (BD Biosciences, Franklin Lakes, NJ, USA). Time lapse imaging was performed at 10 s intervals (Fig. S1B–C).

2. Image processing

To extract the cell contour, we used GFP::PH$^{PLC1\delta1}$-expressing cells and constructed a series of image processing algorithms written in C (Fig. S2A–C). Microscopy images were obtained in 14 bit, where 1 pixel corresponded to ~0.165 μm. Before our algorithms were applied, by using IPLab software, the dynamic range of each frame was normalized, the frames were converted from 14 bit to 8 bit, and the resultant images were saved as TIFF files. The overall strategy for our image processing was as follows: Step 1) a smoothing filter was applied to the image for noise reduction; Step 2) image binarization was performed by picking up pixels with a strong intensity; Step 3) noise that did not form the cell contour was removed; Step 4) cell contours were smoothened; and Step 5) the boundary between the cell contour and cytoplasmic regions was traced. A detailed description of each of these processes is described below.

Step 1) Microscopic images were smoothed with a 3 × 3 averaging filter [6], resulting in a signal intensity for each pixel that was averaged by its 3 × 3 neighboring pixels.

Step 2) Image binarization was performed with a kind of local thresholding [6]. We picked up pixels brighter than their neighboring pixels as follows. The intensity of a pixel of interest (**a**) was compared with that of its neighboring pixels (Fig. S2B). The neighboring pixels ($\mathbf{b}_{1\text{-}2}$-$\mathbf{e}_{1\text{-}2}$) were located at a distance of 8 pixels from pixel **a**. If the intensity of **a** was larger by 10 in the 256-level (8-bit) image (0: white (strongest fluorescence signal), 255: black) than that of $\mathbf{b}_1$ and $\mathbf{b}_2$, pixel **a** was picked up. A similar

decision was performed for the comparison of the intensity of **a** with that of $\mathbf{c}_{1\text{-}2}$, $\mathbf{d}_{1\text{-}2}$, or $\mathbf{e}_{1\text{-}2}$. Note that the 8 pixel distance was a comparable or larger value to the expected width of the cell contour, allowing us to extract pixels of the cell contour.

Step 3) The picked-up pixels were grouped by their connectivity. In general, the area of a group that forms the cell contour was larger than that of the other groups that form noise. Thus, noise could be removed by erasing groups with a smaller area than 50 pixels. The connected components were extracted as previously described [6].

Step 4) The cell contour was smoothened with a combination of erosion and dilation algorithms [6]. Erosion followed by dilation is called "opening," and this smoothes contours and eliminates sharp peaks [6]. Dilation followed by erosion is called "closing," and this smoothes contours and fuses narrow breaks [6]. We performed opening and closing with a 1 and 3 pixel(s) resolution, respectively. Here the pixels that form the cell contour are called "CCPs" (Cell Contour Pixels) (Fig. S2A, the 2nd image).

Step 5) A boundary-following algorithm was used to trace the boundary between CCPs and cytoplasmic regions (Fig. S2C) [6]. The obtained pixels forming the boundary are called "BP1s" (Boundary Pixels) (Fig. S2C).

3. Quantification of cell shape

Quantification procedures

Using CCPs and BP1s, we quantified cell shape parameters, including the values of the *r*-*z* coordinate of the cell contour, curvatures (C_m and C_p), and cell volume and surface area (Figs. 1 and S2A, D–F). The overall strategy was: Step 1) a small number of pixels were selected from the BP1s that mimic the cell contour, and their *r*-*z* coordinates were recorded; Step 2) the cell shapes were assumed to be rotationally symmetrical, and the rotational axis was defined; Step 3) C_m was calculated for each selected pixel; Step 4) C_p was calculated for each selected pixel; Step 5) the cell volume and surface area were calculated; and Step 6) the pixels were resampled so they were equally spaced at 41 points. A detailed

description of each of these processes is described below.

Step 1) A subset of BP1 pixels were selected as “BP2 pixels” (BP2s) to mimic the cell contour. By setting the following rules, we selected the BP2s so that a lot or a few pixels were selected around acutely curved regions, e.g., the apex of the cleavage furrow or around obtusely curved regions, respectively (Fig. S2D). The identity number of the selected pixels is presented by “i (i = 1, 2, 3, … N).” If (i-1) and (i) pixels were given, the (i+1) pixel was selected so that the distance between (i+1) and Line1, which runs on (i-1) and (i), became larger than 2 pixels. Around acutely curved regions, the angle between Line1 and Line2, which runs on (i) and (i+1), becomes very small, leading to a large curvature calculation error in Step 3. To avoid such an error, we inserted an additional pixel (h) between (i-1) and (i) if the angle between Line1 and Line2 was smaller than $\pi/2$. This additional pixel satisfied that the angle between Line2 and a line, which runs on (h) and (i), was larger than $\pi/2$. This procedure was done in the clockwise and counter-clockwise directions. The number of BP2s (N) was ~several 10s for each sample.

Step 2) A rotational axis was defined so that sum of the distances between the rotational axis and the BP1s on one side of the rotational axis became equal to that between the rotational axis and the BP1s on the other side (Fig. S2A, the 3rd image). Since the equator of the cells also satisfied this definition, we added another constraint to avoid falsely defining the equator of the cell as a rotational axis, i.e., the cell length along the rotational axis should be larger than 80% of the cell length along the longest axis, which practically avoids this error.

Step 3) The C_m for each selected pixel was defined as $d\theta/ds$, where θ is an angle between a normal vector at the selected pixel and the rotational axis, and s is the arc length of the cell contour. Thus, we measured θ by defining a normal vector for each BP2 (Fig. S2E–F). The normal vector was calculated by using the neighboring BP2s. A circle that runs on a BP2 of interest (i) and 2 sandwiching BP2s (i-1 and i+1) was calculated, and a vector connecting the BP2 of interest (i) to the center of the circle was defined as a normal vector (Fig. S2E).

By using the normal vectors of the BP2s, we then calculated C_m. First, we

calculated the crossing points for each pair of 2 neighboring normal vectors (Fig. S2F). The curvature ($d\theta/ds$) of an arc sandwiched between the 2 neighboring normal vectors is basically equivalent to the reciprocal of the distance between the crossing point and the arc.

Step 4) The C_p for each BP2 was determined by using the rotational axis and the normal vectors for each BP2. A crossing point between the rotational axis and the normal vector for a BP2 was calculated, and the C_p for the BP2 was defined as the reciprocal of the distance between the BP2 and the crossing point. In general, it is difficult to accurately calculate C_p for BP2s near the rotational axis because slight fluctuations of the *in vivo* cell shape or slight calculation errors of the normal vectors are amplified. Thus, it should be noted that the calculated values of C_p near the rotational axis, which coincide with s = 0.0–0.2, have larger errors.

Step 5) To measure the cell volume and surface area, we first separated a cell into 4 quadrants by its rotational axis and cell equator (Fig. S2A, the final image, q1–4). The cell equator was defined as the position of the cleavage furrow or the cell center along the rotational axis for the early phases when no distinct cleavage furrow had yet emerged. The cell volume and surface area were independently calculated for each quadrant. In this calculation, we approximated the contour as a broken line connecting the BP2s.

Step 6) The BP2s were resampled as 41 equally spaced points, "BP3s," on the broken line connecting the BP2s. Accordingly, the *r-z* coordinates and the curvatures of the BP3s were also determined by using those of the BP2s by linear interpolation of the broken line. As mentioned in Step 1, the BP2s were selected in 2 directions, clockwise and counter-clockwise, and the curvatures derived from both directions were averaged.

<u>Data analyses</u>

Utilizing the methods described above, the values of the *r-z* coordinates, curvatures, pole-to-furrow distances, and cell volume and surface area were measured from 14 or 6 embryos in the wild-type or the *zen-4 ts* mutant cells, respectively. 14.5μm length was normalized to 1 unit. We defined that 1.0 unit length = 14.5 μm, 1.0 unit curvature is 1/14.5 μm = 0.0691 μm^{-1}, 1.0 unit volume = $4 / 3 \times \pi \times 14.5^3 = 1.27 \times 10^4$ μm^3, and 1.0 unit area =

$4 \times \pi \times 14.5^2 = 2.63 \times 10^3$ μm^2. In Figure 1C–F, the values were averaged for each time point, where the time when the furrow radius reached a unit length of 0.5 was defined as 0 s (Fig. 1C). In Figures 2B, 4A–B, S3A–D, S4D, S10A–B, and S11, the values were binned by every 0.1 furrow radius. In Figure S11, after the binning described above, the values for the cell surface area were normalized by multiplying the area [unit area] by (cell volume [unit volume])$^{-2/3}$. In Figure 2B, the values were normalized by multiplying the length [unit length] by (cell volume [unit volume])$^{-1/3}$, and binning was then performed by every 0.1 furrow radius. The P value at $s = 0.7$ in Figure 4B was calculated with Student's t test.

4. Construction and analyses of the bending model

4-1. General assumption

We constructed a model of cytokinesis on the basis of a theory for the bending elasticity of the cell surface, which has been applied to explain the shapes of red blood cells and liposomes [7,8,9,10]. In addition, bending elasticity has been also considered in cytokinesis, mitotic cell rounding, etc. [11,12,13,14,15]. According to previous studies, mathematical models based on bending elasticity accurately reproduce the biconcave-disk shape of red blood cells and their shape transitions during capillary flow [8,9]. Moreover, the various shapes of liposomes (prolate, oblate, stomatocyte, etc.) and their shape transitions by cytoskeletal proteins are also reproduced by mathematical models based on bending elasticity [7,10]. The major assumption of these mathematical models is that these objects favor shapes with global or local minimums of bending energy of the whole surface under the constraints that the volume and surface area of the objects are given. On the basis of this theory, we assumed that the bending energy for 1 surface area unit of a cell (e) was described as:

$$e = \frac{1}{2}K_c\left(C_p + C_m - C_o\right)^2 + K_g C_p C_m \qquad \text{(eq. 1)}$$

Here, K_c and K_g are the bending modulus and the Gaussian bending modulus, respectively.

K_c and K_g represent the elastic properties of the cell surface that reflect the status of the actin-based cytoskeleton. In other words, they are indexes of cell surface stiffness. C_m and C_p are the curvature along the meridians and along the parallels of latitude, respectively. C_o is spontaneous curvature. Spontaneous curvature should be considered if the surface of a cell intrinsically favors a bent plane, but not a flat one; however, in this study, we assumed that C_o was zero. The total bending energy (E) of the whole cell surface was given as:

$$E = \int e dA = \int \left\{ \frac{1}{2} K_c \left(C_p + C_m - C_o \right)^2 + K_g C_p C_m \right\} dA \qquad \text{(eq. 2)}$$

Here, A is the surface area of a cell. Analogous to liposomes, we assumed that cells transform their shapes while satisfying global or local minimums of bending energy during cytokinesis under the constraints of cell volume and surface area. If we defined G as:

$$G = E + PV + TA \qquad \text{(eq. 3)}$$

, where E is the bending energy shown in equation 2, V is the cell volume, A is the cell surface area, and P and T are Lagrange multipliers, the conditions that cell shapes should satisfy could be derived from the variation $\delta G = 0$. P and T can be interpreted as the difference of pressure between the outside and inside of cells and surface tension in a physical context, respectively. We assumed that cell shapes to be rotationally symmetrical. The coordinates are defined as shown in Figure S4A. For these coordinates, C_m is $d\theta/ds$ and C_p is $\sin\theta/r$. Finally, we introduced the contractile ring into equation 3 as a constraint of furrow radius only on the furrow, as described by Umeda *et al.* [10]. To simplify model construction, we assumed that cell shapes were symmetric to the equatorial plane where the contractile ring is located (Fig. S4A–B). Here we defined H as:

$$H = E + PV + TA + 2\pi r\gamma\Big|_{t=t_1} \qquad \text{(eq. 4)}$$

, where γ is a Lagrange multiplier and the position $t = t_1$ corresponds to that of the contractile ring. γ can be interpreted as the line tension/constriction force generated by the contractile ring. When we assume that the cell contour is smooth at the cell pole and the equatorial plane, we can conclude that $\theta(t_0) = 0$ and $\theta(t_1) = \pi/2$.

The variation of equation 4 is calculated as:

$$\delta H = \overline{H} - H \qquad \text{(eq. 5)}$$

, where $\overline{H}$ was calculated for a shape with small geometric fluctuations. A shape with these small fluctuations was described as:

$$\bar{r} = r + \eta \sin\theta + \xi \cos\theta$$

$$\bar{z} = z - \eta \cos\theta + \xi \sin\theta \qquad \text{(eq. 6)}$$

, where η and ξ are the functions of t to provide the small fluctuations in the normal direction and at a tangent to the cell surface, respectively.

4-2. In the case when K_c is spatially constant

If we assumed that K_c, K_g, C_o, and P are spatially constant (Fig. S4B), the variation of equation 5, $\delta H = 0$, led to the following Euler-Lagrange equation:

$$\frac{K_c}{r}\frac{d}{dt}\left\{r\frac{d}{dt}\left(2H - C_o\right)\right\} + K_c\left(2H - C_o\right)\left(2H^2 - 2K + C_o H\right) - 2TH - P = 0 \quad \text{(eq. 7)}$$

, which corresponds the force balance in the perpendicular direction to the cell surface. H and K are the mean curvature ($H = (1/2)\ (C_m + C_p)$) and the Gaussian curvature ($K = C_m\ C_p$), respectively. K_g does not appear in this equation or in the boundary conditions shown later. Note that this equation is essentially the same as previously described [10]. The variation of equation 5 also provided following boundary conditions:

$$2K_c r \left.\frac{dH}{dt}\right|_{t=0} = 0 \qquad \text{(eq. 8)}$$

$$2K_c r \left.\frac{dH}{dt}\right|_{t=t_1} + \gamma = 0 \qquad \text{(eq. 9)}$$

Equation 9 represents the force balance at the position of the contractile ring. Shapes satisfying equations 7–9 and the geometric constraints were calculated by solving the following ordinary differential equations by using the relaxation method [16]:

$$\frac{du}{ds} = t_1\left\{- K_c r\left(2H - C_o\right)\left(2H^2 - 2K + C_o H\right) + 2THr + Pr\right\}$$

$$\frac{dH}{ds} = t_1\left(\frac{u}{2K_c r}\right)$$

$$\frac{d\theta}{ds} = t_1\left(2H - \frac{2\sin\theta}{r}\right)$$

$$\frac{dr}{ds} = t_1 \cos\theta$$

$$\frac{dz}{ds} = t_1 \sin\theta$$

$$\frac{dT}{ds} = 0$$

$$\frac{dP}{ds} = 0$$

$$\frac{dA}{ds} = t_1 2\pi r$$

$$\frac{dV}{ds} = t_1 \pi r^2 \sin\theta$$

$$\frac{dt_1}{ds} = 0$$

Here, t is replaced by s (Fig. S4A. Where t = 0, s = 0. Where $t = t_1$, s = 1). u is $K_c r \frac{d}{dt}\left(2H - C_o\right)$. The boundary conditions were as follows:

at position $s = 0$,

$$r = 0,\ z = 0,\ \theta = 0,\ A = 0,\ V = 0,\ u = 0;$$

at position $s = 1$,

$$\theta = \frac{\pi}{2},\ A = \frac{A_0}{2},\ V = \frac{V_0}{2},\ u = -\gamma\ .$$

Here, A_0 and V_0 were given, while the boundary conditions u = 0 and u = $-\gamma$ were obtained from equations 8 and 9, respectively.

4-2-1. Calculation of the minimum furrow radii and pole-to-furrow distances

Shapes were calculated with the constraints that A and V were given from the *in vivo*

measured values. In the case when A and V in the wild-type cells at each furrow radius were applied, we calculated the relationship between the magnitude of the contractile ring force and furrow radii or between the magnitude of the contractile ring force and pole-to-furrow distances (Fig. S4C). As the contractile ring force increased, the values of the furrow radii and the pole-to-furrow distances became smaller. Finally, when we applied A and V derived from the *in vivo* cell shapes with a furrow radius < 0.1, the furrow radius became almost 0. Conversely, when we applied A and V derived from the *in vivo* cell shapes with a furrow radius > 0.1, the values of the furrow radii and the pole-to-furrow distances reached a plateau, and the former never became 0 because of the geometric constraints of A and V. Thus, under a given condition of A and V, we were able to determine the minimum furrow radius and pole-to-furrow distance that should be achieved for a contractile ring force = ∞. In this condition, since P and T also became ∞, the 1st and 2nd elements in equation 7 were negligible, resulting in the equation $2TH + P = 0$. The solution of this equation was a sphere (Fig. S4D, contractile ring force (Force) = ∞ (blue)). The calculated shapes, even under Force = ∞ or smaller forces, still had slight differences with that of the *in vivo* cells (Fig. S4D). In Figure 2B, we calculated and plotted the minimum furrow radii and pole-to-furrow distances under various conditions with various values of A and V, with or without contractile ring force.

<u>4-2-2. Calculation of the magnitude of the contractile ring force required to achieve a given furrow radius</u>

In Figure 4E, by using the relationship between the magnitude of the contractile ring force and furrow radii (Section 4-2-1 and Fig. S4C), we determined the magnitude of the contractile ring force required to achieve the *in vivo* furrow radii in the wild-type cells.

<u>4-3. In the case when K_c is not spatially constant</u>

Next, we examined the case when K_c is not spatially constant. In other words, K_c is a function of the cell surface position s. We further assumed that C_o is not spatially constant, and K_g and P are spatially constant. Consequently the variation of equation 5 led to the

following Euler-Lagrange equation:

$$\frac{1}{r}\frac{d}{dt}\left[r\frac{d}{dt}\{K_c(2H-C_o)\}\right]+K_c(2H-C_o)(2H^2-2K+C_oH)-2TH-P=0 \quad \text{(eq. 10)}$$

K_g does not appear in either this equation or in the following boundary conditions. The boundary conditions were as follows:

$$r\frac{d}{dt}\{K_c(2H-C_o)\}\bigg|_{t=0}=0 \qquad \text{(eq. 11)}$$

$$r\frac{d}{dt}\{K_c(2H-C_o)\}\bigg|_{t=t_1}+\gamma=0 \qquad \text{(eq. 12)}$$

Furthermore, an additional equation that represents force balance in a parallel direction to the cell surface was obtained:

$$2K_c(2H-C_o)\frac{dH}{dt}-\frac{d}{dt}\left\{\frac{K_c}{2}(2H-C_o)^2\right\}-\frac{dT}{dt}=0 \qquad \text{(eq. 13)}$$

In the present study, we assumed that C_o is spatially constant, resulting in the following equation:

$$\frac{dT}{dt}=-\frac{1}{2}(2H-C_o)^2\frac{dK_c}{dt} \qquad \text{(eq. 14)}$$

Under a given spatial distribution of K_c, we obtained cell shapes by solving the following ordinary differential equations.

$$\frac{du}{ds}=t_1\{-K_cr(2H-C_o)(2H^2-2K+C_oH)+2THr+Pr\}$$

$$\frac{dH}{ds}=t_1\left\{\frac{u}{2K_cr}-\frac{(2H-C_o)}{2K_ct_1}\frac{dK_c}{ds}\right\}$$

$$\frac{d\theta}{ds}=t_1\left(2H-\frac{2\sin\theta}{r}\right)$$

$$\frac{dr}{ds}=t_1\cos\theta$$

$$\frac{dz}{ds}=t_1\sin\theta$$

$$\frac{dT}{ds} = -\frac{1}{2}(2H - C_o)^2 \frac{dK_c}{dt}$$

$$\frac{dP}{ds} = 0$$

$$\frac{dA}{ds} = t_1 2\pi r$$

$$\frac{dV}{ds} = t_1 \pi r^2 \sin\theta$$

$$\frac{dt_1}{ds} = 0$$

Here, u is $r\frac{d}{dt}\{K_c(2H - C_o)\}$. Boundary conditions were the same as those in the case when K_c is constant.

5. Estimation of the spatio-temporal changes in the bending modulus

The spatial distributions of the bending modulus could not be analytically estimated; thus, we numerically estimated the spatial distributions of K_c by repeated improvement of the distributions. Importantly, the estimation was performed in a dimensionless manner because the focus of the present study was the relative spatial differences of the bending modulus.

5-1. Overview of the method

Step 1) An initial spatial distribution of K_c was arbitrarily provided. The value of the contractile ring force was also given. A shape with minimum bending energy was then calculated as shown in Section 4 (Construction and analyses of the bending model).

Step 2) The difference between the calculated and *in vivo* shapes was evaluated with the cost function J:

$$J = \sum_{1}^{M} \left\{ (z - \bar{z})^2 + (r - \bar{r})^2 \right\} \quad \text{(eq. 15)}$$

Here, z and r are the values of the coordinates of the calculated shape for each point on the cell contour. $\bar{z}$ and $\bar{r}$ are the values of the coordinates of the *in vivo* shape for each point on the cell contour, while M is the number of points on the cell contour.

Step 3) The spatial distribution of K_c was improved to decrease the value of J. The improvement was repeatedly performed with the quasi-Newton method [16]; consequently, we obtained the spatial distribution of K_c that minimized the value of J.

5-2. Methods in detail

We assessed 3 methods, all of which were essentially based on the method described in Section 5-1.

a. Method 1

In Step 1) of Section 5-1, the K_c values for each point were independently given. In Step 3), the $\partial A/\partial K_c$ values for each point were calculated by numerical differentiation, and the optimal K_c values for each point were identified with the quasi-Newton method. However, this method did not work well, that is, the calculated spatial distributions of K_c were affected by the initial ones given in Step 1) and were not smooth (data not shown). This was a result of over-fitting due to the high freedom of this problem.

b. Method 2

To decrease the freedom, we presented the spatial distribution of K_c by using cosine curves as:

$$K_{c\_i} = B + \sum_{n=1}^{N} b_n \cos\left(\pi n \frac{i-1}{M-1} \right), \quad (i = 1,,,,,, M) \quad \text{(eq. 16)}$$

By using cosine curves, the spatial distribution of K_c was expected to be smooth even at the position $s = 0$ and $s = 1$. To set the overall scale of the K_c values, B was introduced and set as 1.0. In Step 1), the values of b_n ($n = 1{\sim}N$) were given. In Step 3), the values

of $\partial J/\partial b_n$ ($n = 1\sim N$) were calculated by numerical differentiation, and the optimal values of b_n were calculated with the quasi-Newton method.

We defined a threshold value of J/M to determine whether the calculated b_n acceptably reproduced the *in vivo* cell shapes. We set the threshold value $J/M = 0.0001$, indicating that the average distance between the computationally calculated and *in vivo* shapes for each point is $0.0001^{1/2} = 0.01$ in the scale of Figure 1F. This difference is visually almost unrecognizable.

n was set as the minimum value to satisfy $J/M < 0.0001$. A large n could almost always satisfy $J/M < 0.0001$, but the calculated b_n was affected by the initial value given in Step 1). In 96% of the samples, the minimum value of n that satisfied $J/M < 0.0001$ was less than 5. Under the condition where the constriction force = 0, 88% of samples yielded b_n that was not affected by the initial value in Step 1) and satisfied $J/M < 0.0001$. The percentages were 93, 60, 20, and 7% for a constriction force = 1.0, 10, 50, and 100, respectively; thus, under larger constriction forces, the percentage was rapidly decreased. The observation that the optimal distribution of b_n was not efficiently obtained under higher constriction forces may be due to the high constraint for the spatial distribution of K_c because of the use of cosine curves. The calculated spatial distribution of K_c for a constriction force = 0 is shown in Figure S5. In the later phase of cytokinesis, the K_c around the furrow became very small and almost 0. This overall pattern of K_c was similar to that observed in Figure 5A, which was obtained by Method 3. We occasionally generated b_n whose K_c values around the furrow were less than 0, although K_c should be >0 in the physical context. This result may imply that the control of the K_c value on a very small scale (nearly 0) is critical for shape calculation. To avoid $K_c < 0$ and efficiently obtain K_c values under larger constriction forces, we assessed another method.

c. Method 3

To decrease the freedom, we introduced a cost for the spatial smoothness of K_c into the cost function J. When the cost for the spatial smoothness of K_c was formulated, the

value of κ = log (K_c) was used to avoid K_c values < 0, as K_c should be >0 in the physical context. We also introduced a cost to set the scale of the K_c values.

$$J = \sum_{1}^{M} \left\{ (z - \bar{z})^2 + (r - \bar{r})^2 \right\}$$

$$+ \omega_1 \left[\sum_{2}^{M-1} \left(2\kappa|_m - \kappa|_{m-1} - \kappa|_{m+1}\right)^2 + \left(2\kappa|_1 - 2\kappa|_2\right)^2 + \left(2\kappa|_M - 2\kappa|_{M-1}\right)^2 \right]$$

$$+ \omega_2 \left[\frac{1}{M} \sum_{1}^{M} \kappa - \bar{\kappa} \right]^2 \qquad \text{(eq. 17)}$$

Here, the 2nd element is the cost for the spatial smoothness of κ, and the 3rd element is the cost to set the scale of κ to a given value $\bar{\kappa}$. ω_1 and ω_2 are the weights for each element. In Step 3), the values of $\partial J/\partial \kappa$ for each point on the cell contour were calculated by numerical differentiation, and the optimal values of κ for each point were calculated with the quasi-Newton method.

Here, we also defined a threshold value of J/M to determine whether the calculated spatial distribution of κ acceptably reproduced the *in vivo* cell shapes. We set the threshold value J/M = 0.0001. The value of ω_2 affected the scale of κ, but essentially not the spatial patterns of κ. The value of ω_2 was set as 1.0 so that the values of (κ - $\bar{\kappa}$) for the output κ was <0.01. In the present study, $\bar{\kappa}$ was set as 1.0. The value of ω_1 was set as 0.05. Larger ω_1 values caused the calculated distributions of κ to not satisfy J/M < 0.0001. The percentages of such samples for a contractile ring force = 0 were <7% and 20% for ω_1 = 0.05 and ω_1 = 1.0, respectively. Conversely, smaller ω_1 caused the calculated spatial distributions of κ to be affected by the initial values given in Step 1). The percentages of such samples were <7% and 27% for ω_1 = 0.05 and ω_1 = 0.001, respectively. The calculated spatial distributions of κ were similar under conditions with 1.0 > ω_1 > 0.01 (Fig. S6).

In the presence of the contractile ring force, the percentages of the successful estimation of κ were changed to >93, >93, 93, 80, and 47% for a contractile ring force = 0, 1.0, 10, 50, and 100, respectively. These changes may be due to the narrower range

of optimal magnitudes for the contractile ring force, which appears to be from 0 to ~30, as suggested from Figure 4E.

5-3. Data analyses of the estimated spatial distributions of K_c

Spatial distributions of K_c were estimated in 4 embryos from the wild-type and *zen-4 ts* cells. Figures S6B and S7B show examples of the raw data. In Figures 3, 4C, 5A, S5, S8, and S9, the estimated values were binned by every 0.1 furrow radius. The majority of the *zen-4 ts* cells arrested the furrow at a furrow radius of 0.6–0.5; thus, the values in the *zen-4 ts* cells for a furrow radius < 0.5 were not estimated. N = 29, 59 (0.9–0.8), 36, 73 (0.8–0.7), 27, 92 (0.7–0.6), 30, 124 (0.6–0.5), 28, not estimated (n.e.) (0.5–0.4), 17, n.e. (0.4–0.3), 16, n.e. (0.3–0.2), 17, n.e. (0.2–0.1), and 8, n.e. (0.1–0.0) for each furrow radius (in parentheses) under contractile ring force (Force) = 0 in the wild-type or *zen-4 ts* cells, respectively (Figs. 5A and S9A, Force = 0). N = 19 (0.9–0.8), 38 (0.8–0.7), 28 (0.7–0.6), 29 (0.6–0.5), 28 (0.5–0.4), 18 (0.4–0.3), 17 (0.3–0.2), 18 (0.2–0.1), and 6 (0.1–0.0) for each furrow radius (in parentheses) under Force = 0 in the wild-type cells (Fig. S5). N = 30 (0.9–0.8), 38 (0.8–0.7), 28 (0.7–0.6), 30 (0.6–0.5), 28 (0.5–0.4), 18 (0.4–0.3), 18 (0.3–0.2), 13 (0.2–0.1), and 5 (0.1–0.0) for each furrow radius (parenthesis) under Force = 5 in the wild-type cells (Fig. 3). N = 26, 70 (0.9–0.8), 37, 82 (0.8–0.7), 27, 93 (0.7–0.6), 30, 117 (0.6–0.5), 28, n.e. (0.5–0.4), 18, n.e. (0.4–0.3), 19, n.e. (0.3–0.2), 10, n.e. (0.2–0.1), and 0, n.e. (0.1–0.0) for each furrow radius (in parentheses) under Force = 20 in the wild-type or *zen-4 ts* cells, respectively (Figs. 4C, S8, and S9A, Force = 20). N = 19 (0.9–0.8), 29 (0.8–0.7), 27 (0.7–0.6), 29 (0.6–0.5), 24 (0.5–0.4), 16 (0.4–0.3), 14 (0.3–0.2), 8 (0.2–0.1), and 0 (0.1–0.0) for each furrow radius (in parentheses) under Force = 50 in the wild-type cells (Figs. S8 and S9B). Note that we often failed to obtain the spatial distributions of K_c under a stronger contractile ring force at smaller furrow radii, as described in Section 5-2. The P value at $s = 1.0$ in Figure 4C was calculated with Student's t test.

5-4. Analyses of impaired furrow ingression in *zen-4 ts* cells

In Figure 4D, we assessed how the failure in the local reduction of K_c around the furrow in

the *zen-4 ts* cells (Fig. 4C) affected furrow ingression. In other words, we addressed the possible disadvantages of the spatial distributions of K_c in the *zen-4 ts* cells on furrow ingression compared with those in the wild-type cells. The spatial distributions of K_c in the *zen-4 ts* and wild-type cells at each furrow radius were applied to calculate cell shapes under a given cell volume and surface area derived from the *zen-4 ts* cells at each furrow radius. For example, in the case that we employed the cell volume and surface area at a furrow radius = 0.6–0.5 in the *zen-4 ts* cells, we applied the spatial distributions of K_c at a furrow radius = 0.6–0.5 under Force = 20 estimated for the *zen-4 ts* and wild-type cells (Figs. 4C and S9, Force = 20). We then calculated cell shapes and their furrow radii. Note that the cell volume and surface area used were the averaged values at a furrow radius = 0.6–0.5 in the *zen-4 ts* cells, and the spatial distributions of K_c were not the averaged values in each cell strain, but the values for the individual samples in each cell strain (N = 30 and 117 at a furrow radius = 0.6–0.5 in the wild-type and *zen-4 ts* cells, respectively). When we applied the spatial distributions of K_c estimated in the *zen-4 ts* cells, the calculated furrow radii should be, of course, essentially the same on average to the experimentally measured value (0.6–0.5) in the *zen-4 ts* cells, despite slight errors. In comparison to this calculated furrow radii, the application of the spatial distributions of K_c estimated in the wild-type cells yielded significantly smaller furrow radii (~2% smaller on average) (Fig. 4D, furrow radius = 0.6–0.5; $P < 0.05$, Student's t test). Similar analyses were performed for the spatial distributions of K_c in the *zen-4 ts* and wild-type cells at each furrow radius under the cell volume and surface area derived from the *zen-4 ts* cells at each furrow radius (Fig. 4D, furrow radius = 0.9–0.8 ~ 0.7–0.6. N = 26, 70 (0.9–0.8), 37, 82 (0.8–0.7), and 27, 93 (0.7–0.6), in the wild-type and *zen-4 ts* cells, respectively.). Note that, in Figure 4D, ΔFurrow radius (%) indicates the percentage changes from the furrow radii calculated by applying the spatial distributions of K_c in the *zen-4 ts* cells.

5-5. The absolute value of K_c and the contractile ring force

Our estimation of K_c was performed in a dimensionless manner as shown in Section 5-2. Thus, we did not include any information about the absolute values of K_c and the

constriction force by the contractile ring. The K_c of amoeba cells, red blood cells, and liposomes have been experimentally measured and ranged from 10^{-18} to 10^{-19} J [17,18,19,20,21]. If we assume that 1.0 A.U. of K_c in our analyses corresponds to 2×10^{-18} J [20], 1.0 unit of the contractile ring force in our analyses coincides with ~0.14 pN. A single molecule of a motor protein can exert a force of approximately several pN; thus, only a slight magnitude of the contractile ring force is sufficient to form a furrow.

6. Surface tension model

6-1. Surface tension model with spatially constant surface tension

We tested whether a surface tension-based model could reproduce the *in vivo* cell shapes. We assumed that surface tension was spatially constant, which is analogous to a liquid droplet or a soap bubble [22,23]. In this model, the following equation should be satisfied:

$$2TH + P = 0$$

Here, T is surface tension, H is the mean curvature ($H = 1/2\ (C_m + C_p)$), and P is the difference in pressure between the outside and inside of the cell. Furthermore, T is spatially constant throughout the cell surface. The constriction force derived from the contractile ring was assumed to be generated just on the furrow. Cell shapes were calculated in the surface tension model so that the cell volume and surface area were the same as those quantified in the *in vivo* cells. The calculated cell shape was not consistent with the *in vivo* cell shape at a furrow radius = 0.2–0.1 (Fig. S4D; blue vs. black. Note that the blue shape is the same as that in the bending model under a contractile ring force (Force) = ∞), and any shape satisfying the constraints with cell volume and surface area was not obtained at a furrow radius = 0.1–0.0 (data not shown). To clarify the features of the difference between the *in vivo* and *in silico* shapes, we focused on the relationship between furrow radii and pole-to-furrow distances (Fig. 2B). The plots obtained in the surface tension model were inconsistent with those in the *in vivo* cells (Fig. 2B; green open circles vs. black. Note that the green open circles are the same as those in the bending model under Force = ∞). These

results indicate that this surface tension model cannot reproduce the *in vivo* cell shapes.

<u>6-2. Estimation of surface tension</u>

A surface tension model in which surface tension is assumed to be spatially inconstant has been constructed where the following equation should be satisfied (Fig. S13A) [24]:

$$T_m C_m + T_p C_p + P = 0$$

Here P is the difference in pressure between the outside and inside of a cell and was assumed to be spatially constant. By using this model, spatial distributions of surface tension are known to be analytically estimated by using the curvature C_m and C_p as shown below [24]:

$$T_m = \frac{P}{2C_p}$$

$$T_p = \frac{P}{2C_p}\left(2 - \frac{C_m}{C_p}\right)$$

P was experimentally estimated in previous studies [25]; however, in the present study, P was set as 1.0 because the values of P does not affect the spatial patterns of surface tension. Thus, T_m and T_p for each point on the cell contour could be calculated with the experimentally calculated C_m and C_p. The calculated T_m and T_p values in the wild-type cells are shown in Figure S13B. A peak of T_p around the furrow can be interpreted as being derived from the contractile ring force (Fig. S13B). Around the neighboring region of the furrow, with a higher C_m (Fig. S3B; red arrowheads), T_p was reduced. Such a characteristic feature was also observed in sea urchin embryos [25], and may contribute to the higher C_m region around the neighboring region of the furrow.

We also calculated T_p in the *zen-4 ts* cells (Fig. S14). There were no or only slight differences between the spatio-temporal changes in T_p in the wild-type and *zen-4 ts* cells. These results may be consistent with the observation that the accumulation of myosin II toward the furrow was not defective in the *C. elegans* embryonic *zen-4 ts* cells [26]. Together with our findings, the arrest of furrow ingression in the *zen-4 ts* cells may not be

caused by defects in surface tension.

7. References in supporting information

1. Brenner S (1974) The genetics of Caenorhabditis elegans. Genetics 77: 71-94.
2. Audhya A, Hyndman F, McLeod IX, Maddox AS, Yates JR, 3rd, et al. (2005) A complex containing the Sm protein CAR-1 and the RNA helicase CGH-1 is required for embryonic cytokinesis in Caenorhabditis elegans. J Cell Biol 171: 267-279.
3. Severson AF, Hamill DR, Carter JC, Schumacher J, Bowerman B (2000) The aurora-related kinase AIR-2 recruits ZEN-4/CeMKLP1 to the mitotic spindle at metaphase and is required for cytokinesis. Curr Biol 10: 1162-1171.
4. Edgar LG (1995) Blastomere culture and analysis. Methods Cell Biol 48: 303-321.
5. Shelton CA, Bowerman B (1996) Time-dependent responses to glp-1-mediated inductions in early C. elegans embryos. Development 122: 2043-2050.
6. Gonzalez RC, Woods RE (2007) Digital Image Processing Third edition: Pearson Prentice Hall.
7. Hotani H, Inaba T, Nomura F, Takeda S, Takiguchi K, et al. (2003) Mechanical analyses of morphological and topological transformation of liposomes. Biosystems 71: 93-100.
8. Deuling HJ, Helfrich W (1976) Red blood cell shapes as explained on the basis of curvature elasticity. Biophys J 16: 861-868.
9. Noguchi H, Gompper G (2005) Shape transitions of fluid vesicles and red blood cells in capillary flows. Proc Natl Acad Sci U S A 102: 14159-14164.
10. Umeda T, Suezaki Y, Takiguchi K, Hotani H (2005) Theoretical analysis of opening-up vesicles with single and two holes. Phys Rev E Stat Nonlin Soft Matter Phys 71: 011913.
11. Kunda P, Pelling AE, Liu T, Baum B (2008) Moesin controls cortical rigidity, cell

rounding, and spindle morphogenesis during mitosis. Curr Biol 18: 91-101.

12. Kajita A, Yamamura M, Kohara Y (2002) Physical modeling of the cellular arrangement in C. elegans early embryo: effect of rounding and stiffening of the cells. Genome Inform 13: 224-232.
13. Ujihara Y, Nakamura M, Miyazaki H, Wada S (2010) Proposed spring network cell model based on a minimum energy concept. Ann Biomed Eng 38: 1530-1538.
14. Xiong Y, Rangamani P, Fardin MA, Lipshtat A, Dubin-Thaler B, et al. (2010) Mechanisms controlling cell size and shape during isotropic cell spreading. Biophys J 98: 2136-2146.
15. Forgacs G, Newman SA (2005) Biological Physics of the Developing Embryo. Cambridge: Cambridge University Press.
16. Press WH, Teukolsky SA, Vetterling WT, Flannery BP (1988) Numerical Recipes in C. Cambridge: Cambridge University Press.
17. Reichl EM, Effler JC, Robinson DN (2005) The stress and strain of cytokinesis. Trends Cell Biol 15: 200-206.
18. Betz T, Lenz M, Joanny JF, Sykes C (2009) ATP-dependent mechanics of red blood cells. Proc Natl Acad Sci U S A 106: 15320-15325.
19. Yoon YZ, Hong H, Brown A, Kim DC, Kang DJ, et al. (2009) Flickering analysis of erythrocyte mechanical properties: dependence on oxygenation level, cell shape, and hydration level. Biophys J 97: 1606-1615.
20. Simson R, Wallraff E, Faix J, Niewohner J, Gerisch G, et al. (1998) Membrane bending modulus and adhesion energy of wild-type and mutant cells of Dictyostelium lacking talin or cortexillins. Biophys J 74: 514-522.
21. Sackmann E (1994) The seventh Datta Lecture. Membrane bending energy concept of vesicle- and cell-shapes and shape-transitions. FEBS Lett 346: 3-16.
22. Zhang W, Robinson DN (2005) Balance of actively generated contractile and resistive forces controls cytokinesis dynamics. Proc Natl Acad Sci U S A 102: 7186-7191.
23. Yoneda M, Dan K (1972) Tension at the surface of the dividing sea-urchin egg. J Exp Biol 57: 575-587.

24. Hiramoto Y (1981) Mechanical Properties of Dividing Cells. In: Zimmerman AM, editor. Cellular Dynamics: Mitosis/Cytokinesis. New York: Academic Press. pp. 397-418.
25. Miyoshi H, Satoh SK, Yamada E, Hamaguchi Y (2006) Temporal change in local forces and total force all over the surface of the sea urchin egg during cytokinesis. Cell Motil Cytoskeleton 63: 208-221.
26. Canman JC, Lewellyn L, Laband K, Smerdon SJ, Desai A, et al. (2008) Inhibition of Rac by the GAP activity of centralspindlin is essential for cytokinesis. Science 322: 1543-1546.
