# Supplementary figures and images for: A High-Resolution Shape Fitting and Simulation Demonstrated Equatorial Cell Surface Softening during Cytokinesis and Its Promotive Role in Cytokinesis
Source: PLoS One. 2012 Feb 16;7(2):e31607. doi: 10.1371/journal.pone.0031607 (PMC3281004; doi:10.1371/journal.pone.0031607)

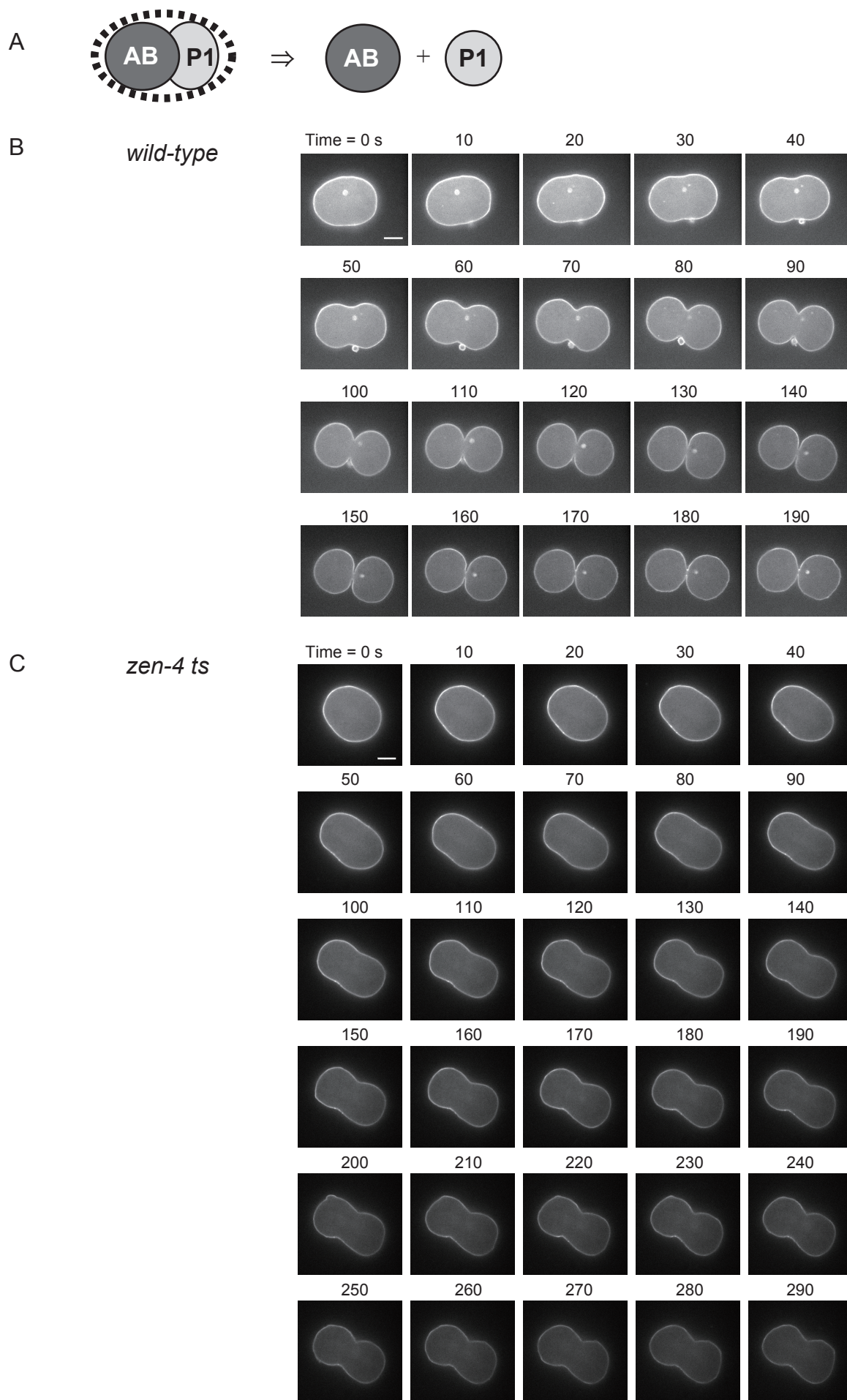

Figure S1

Supplement: Figure S1 — Microscopy images of isolated AB cells. (A) AB cells were isolated from 2-cell stage embryos as described in Section 1 (Materials and manipulation of Caenorhabditis elegans cells). (B) Time-lapse images of a wild-type AB cell expressing GFP::PHPLC1δ1. Scale bar, 10 µm. (C) Time-lapse images of a zen-4 ts mutant AB cell expressing GFP::PHPLC1δ1. Scale bar, 10 µm. (PDF) [file pone.0031607.s002.pdf]

A

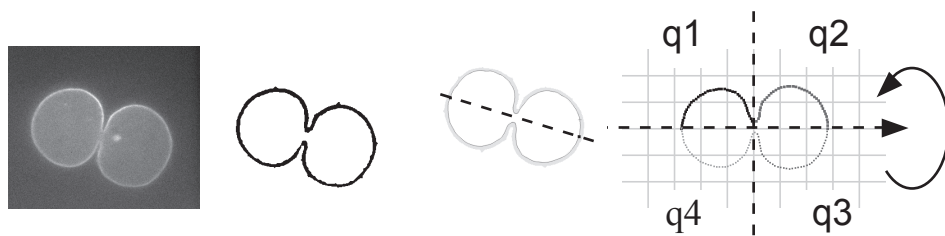

B

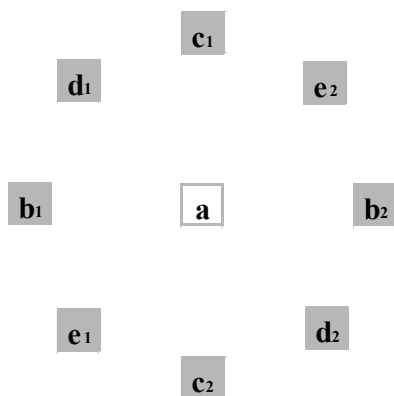

C

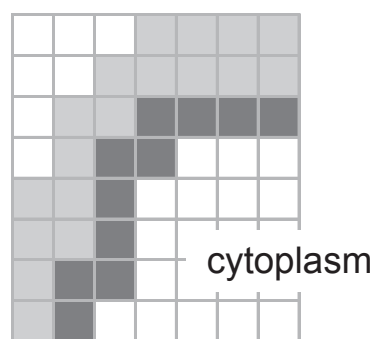

D

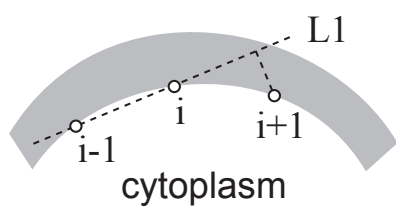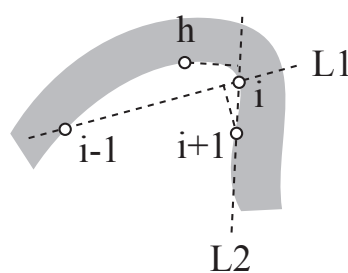

E

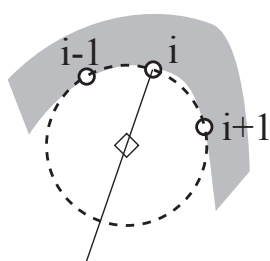

F

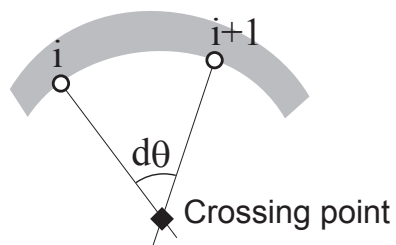

Figure S2

Supplement: Figure S2 — Image processing and quantification of cell shapes. (A) Overview of the procedures. The first panel is a raw microscopy image. The second panel is after the extraction of the CCPs (Cell Contour Pixels). The third panel is the determination of the rotational axis (dashed line). The final panel is the separation of a cell into 4 quadrants (q1–q4) by the rotational axis (horizontal dashed line) and equatorial plane (vertical dashed line). Cell shape parameters, including the values of the r-z coordinates, curvatures, and cell volume and surface area, were independently quantified for each quadrant. (B) Image binarization by local thresholding. The positions of pixel a and pixels b1–2–e1–2 are shown. The intensity of a was compared with that of b1–2–e1–2. See Text S1 for details. (C) Selection of BP1s (Boundary Pixels). CCPs are shown in light and dark gray. BP1s were selected from CCPs along the boundary between CCPs and the cytoplasmic region by a boundary-following algorithm (dark gray). (D) Selection of BP2s. (Left panel) The positional relationship of BP2s (i−1, i, and i+1) is shown. BP2s were selected from BP1s that were located along the boundary between CCPs (gray) and the cytoplasmic region. i+1 was selected so that the distance between Line1 (L1) and i+1 was larger than 2 pixels. (Right panel) In the case for an acutely curved region, where the angle between Line1 (L1) and Line2 (L2) was smaller than π/2 degrees, an additional pixel, h, was inserted. See Text S1 for details. (E) Determination of normal vectors. A circle that runs through i−1, i, and i+1 is shown (dashed circle). The normal vector for i was defined as the solid line that ran through i and the center (diamond) of the circle. (F) Calculation of the curvature Cm. The crossing point (diamond) and the angle dθ between the normal vectors for i and for i+1 are shown. The curvature Cm of the arc sandwiched between i and i+1 was defined as the reciprocal of the distance between the crossing point and th [file pone.0031607.s003.pdf]

A

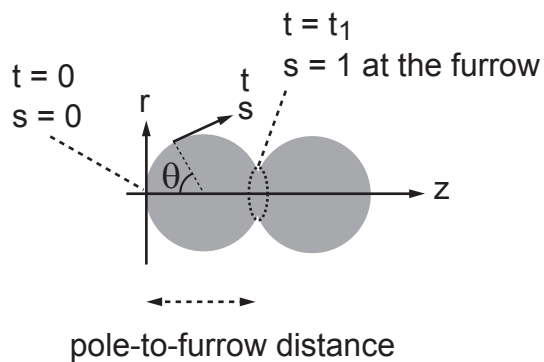

B

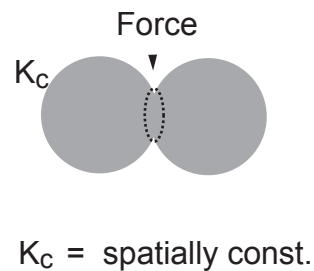

C

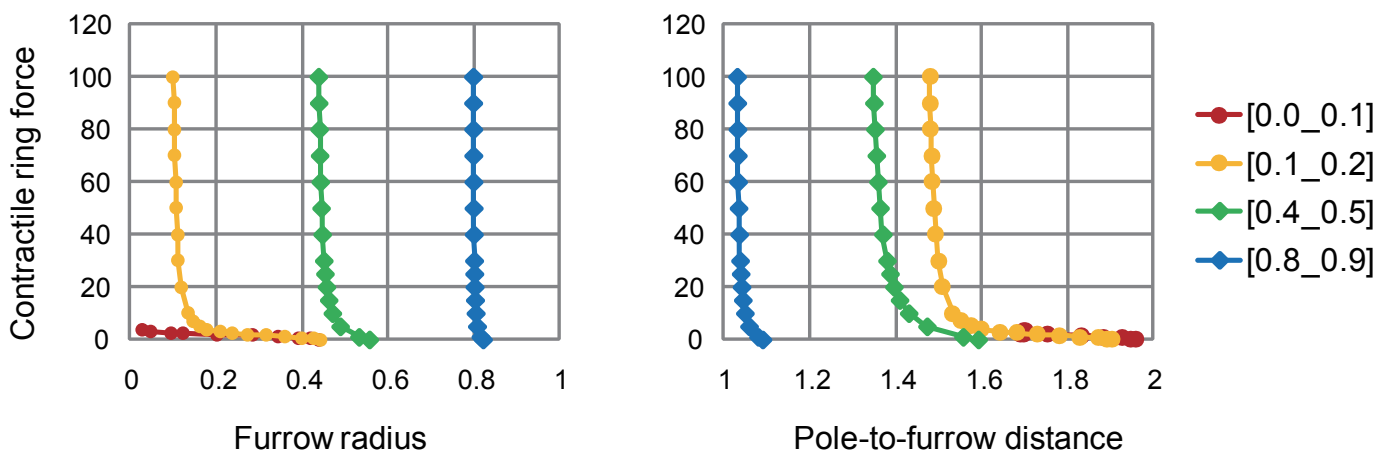

D

Furrow radius = 0.2~0.1

Shape

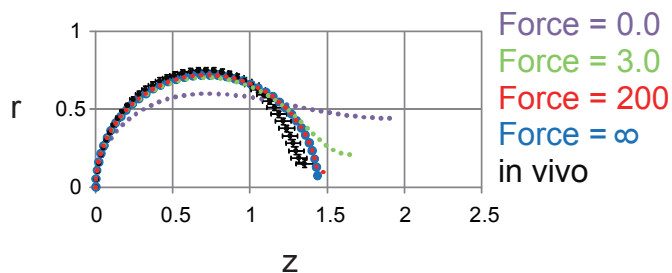Curvature  $C_m$ 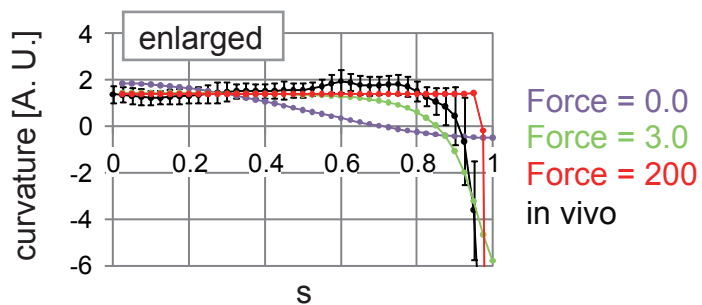

Figure S4

Supplement: Figure S4 — Bending model with spatially constant Kc . (A) Definition of coordinates. The t coordinate starts from a cell pole (t = 0), runs along the cell contour, and ends at the furrow (t = t1). Normalization of the t coordinate by t1 generates the s coordinate. θ is the angle between the rotational axis z and the normal vector of the cell contour. (B) Schematic illustration of the bending model with spatially constant Kc. “Force” indicates the constriction force of the contractile ring. (C) Relationships between the contractile ring forces and furrow radii and between the contractile ring forces and pole-to-furrow distances. In the calculation of shapes, cell volume and surface area were fixed. Both parameters were derived from the average values in the wild-type cells at the presented furrow radii (0.0–0.1, 0.1–0.2, 0.4–0.5, and 0.8–0.9). (D) Comparison of shapes between the wild-type cells and the bending model. The shapes and curvature Cm in the wild-type cells (N = 53) and those obtained in (C) (0.2–0.1) are shown for each value of the force. See the Text S1 for a description of the calculation for the shape under a force = ∞. (PDF) [file pone.0031607.s005.pdf]

Force = 0.0

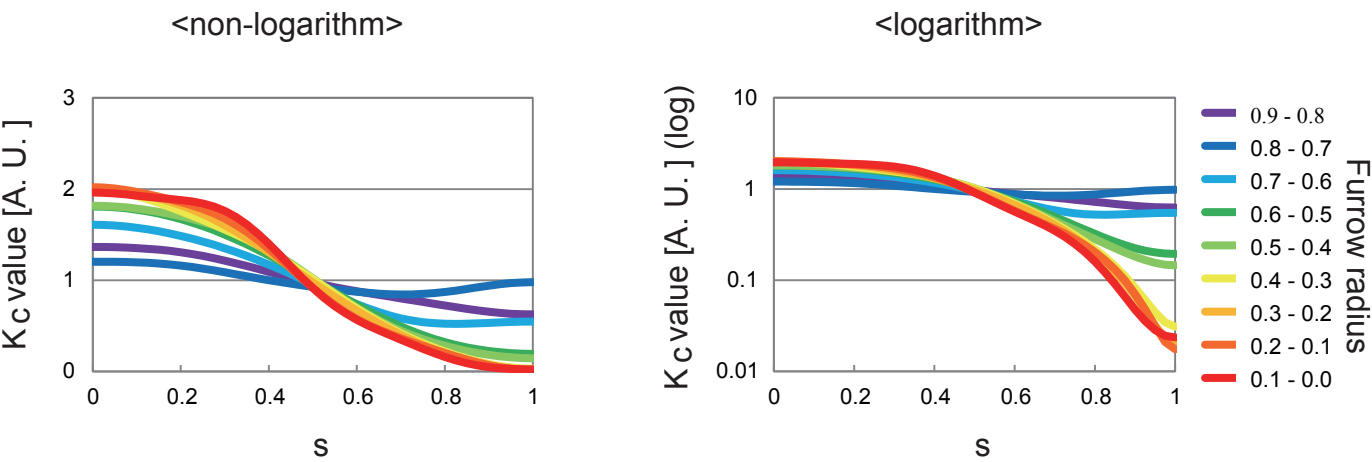

Figure S5

Supplement: Figure S5 — Estimation of the spatio-temporal changes in Kc by Method 2 using cosine curves. Kc values are shown in a non-logarithmic (left panel) or logarithmic manner (right panel). (PDF) [file pone.0031607.s006.pdf]

A

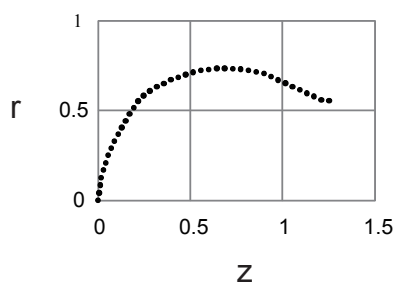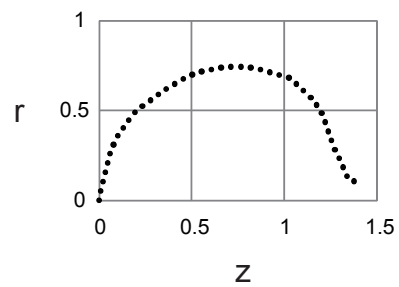

B

 $\omega_1 = 0.01$ 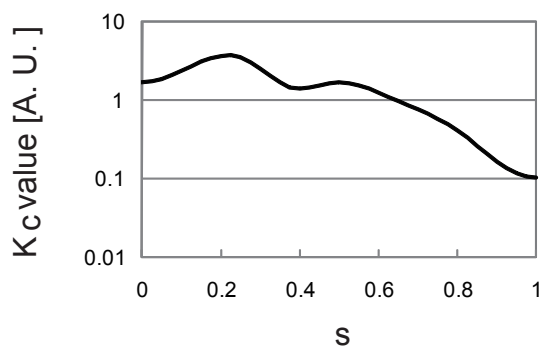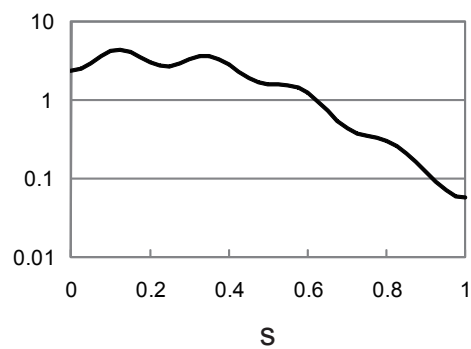 $\omega_1 = 0.05$ 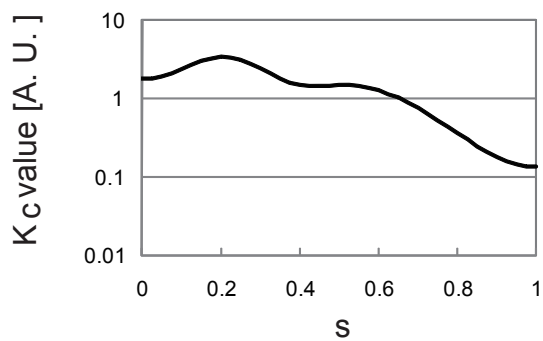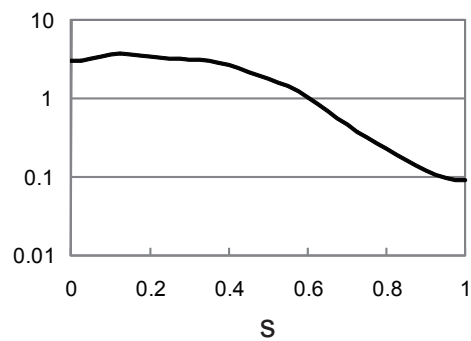 $\omega_1 = 1.0$ 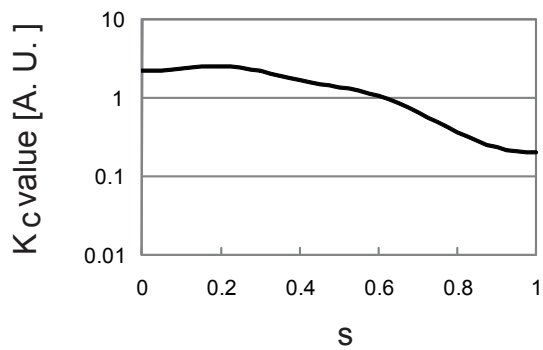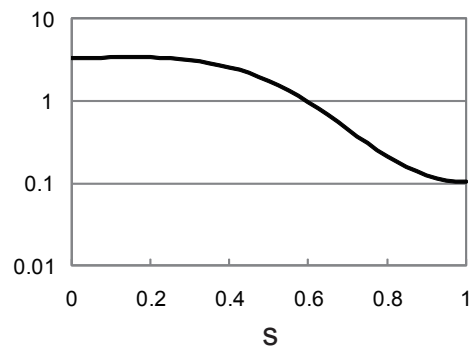

Figure S6

Supplement: Figure S6 — Effect of the weight of the smoothness cost on estimating the spatial changes in Kc by Method 3. (A) Two examples of in vivo cell shapes are shown. (B) Estimated spatial patterns of Kc for the 2 in vivo cell shapes are shown under the different weight (ω1) of the smoothness cost. (PDF) [file pone.0031607.s007.pdf]

A

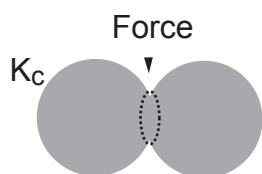

$K_c \neq$  spatially const.

B

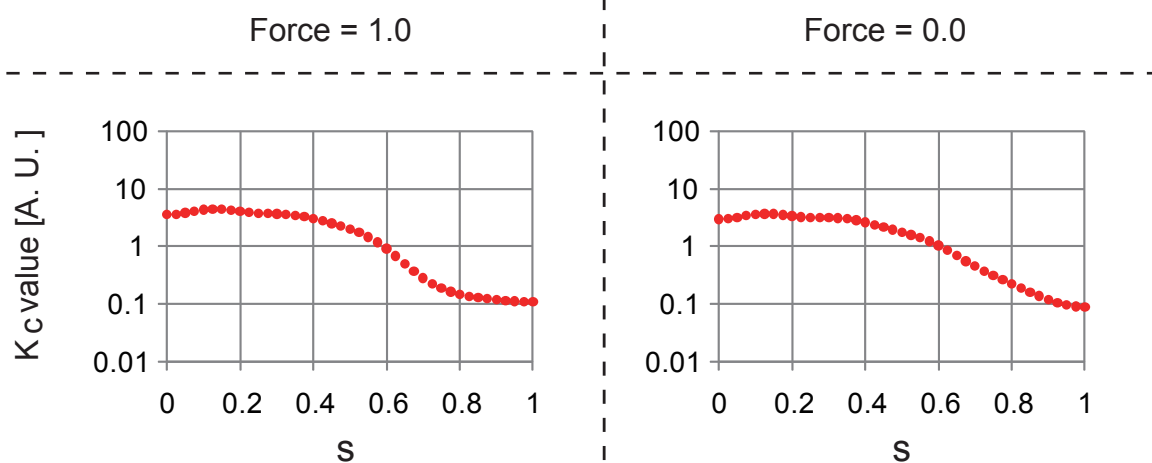

C

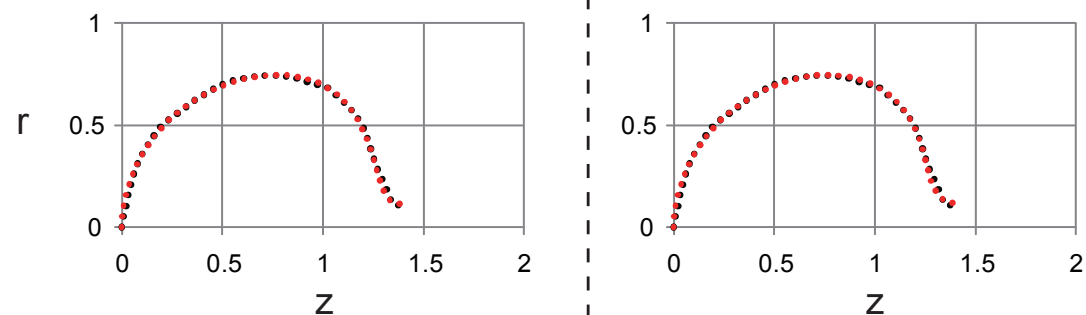

*in vivo*  
*in silico*

D

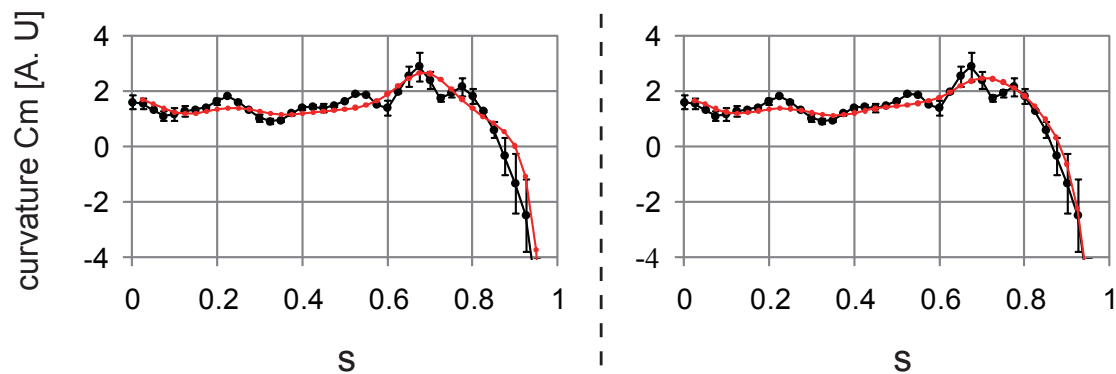

*in vivo*  
*in silico*

Figure S7

Supplement: Figure S7 — Shapes under the estimated spatial patterns of Kc . (A) Schematic illustration of the bending model with spatially inconstant Kc. “Force” indicates the constriction force of the contractile ring. (B) Examples of estimated Kc values in the presence or absence of contractile ring force. (C) Comparison of shapes calculated under the Kc values shown in (B) with the in vivo shapes. The shapes in the model were in good agreement with the in vivo cell shapes. (D) Comparison of the curvature Cm calculated under the Kc values shown in (B) with the in vivo curvature. The higher Cm region around the neighboring regions of the furrow was accurately reproduced under these Kc values, suggesting that the higher Cm was linked to the spatially inconstant distribution of Kc. (PDF) [file pone.0031607.s008.pdf]

Force = 20.0

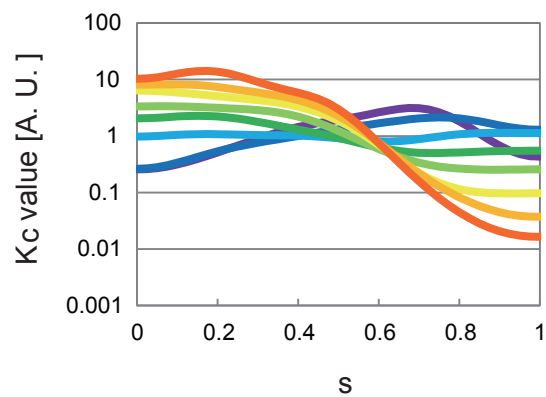

Force = 50.0

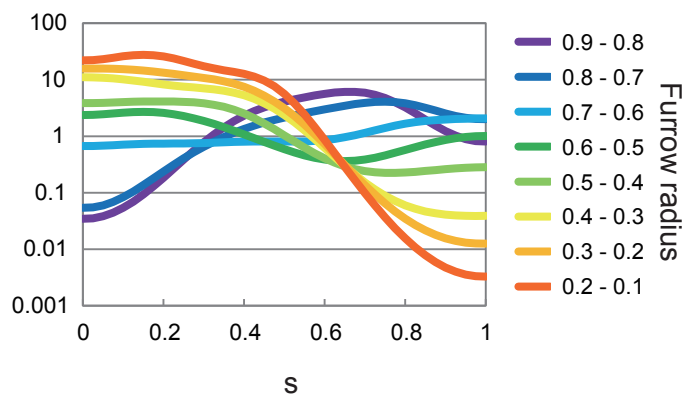

Figure S8

Supplement: Figure S8 — Estimation of the spatio-temporal changes in Kc by Method 3. Kc values estimated under Force = 20.0 (left panel) and = 50.0 (right panel) are shown. (PDF) [file pone.0031607.s009.pdf]

A

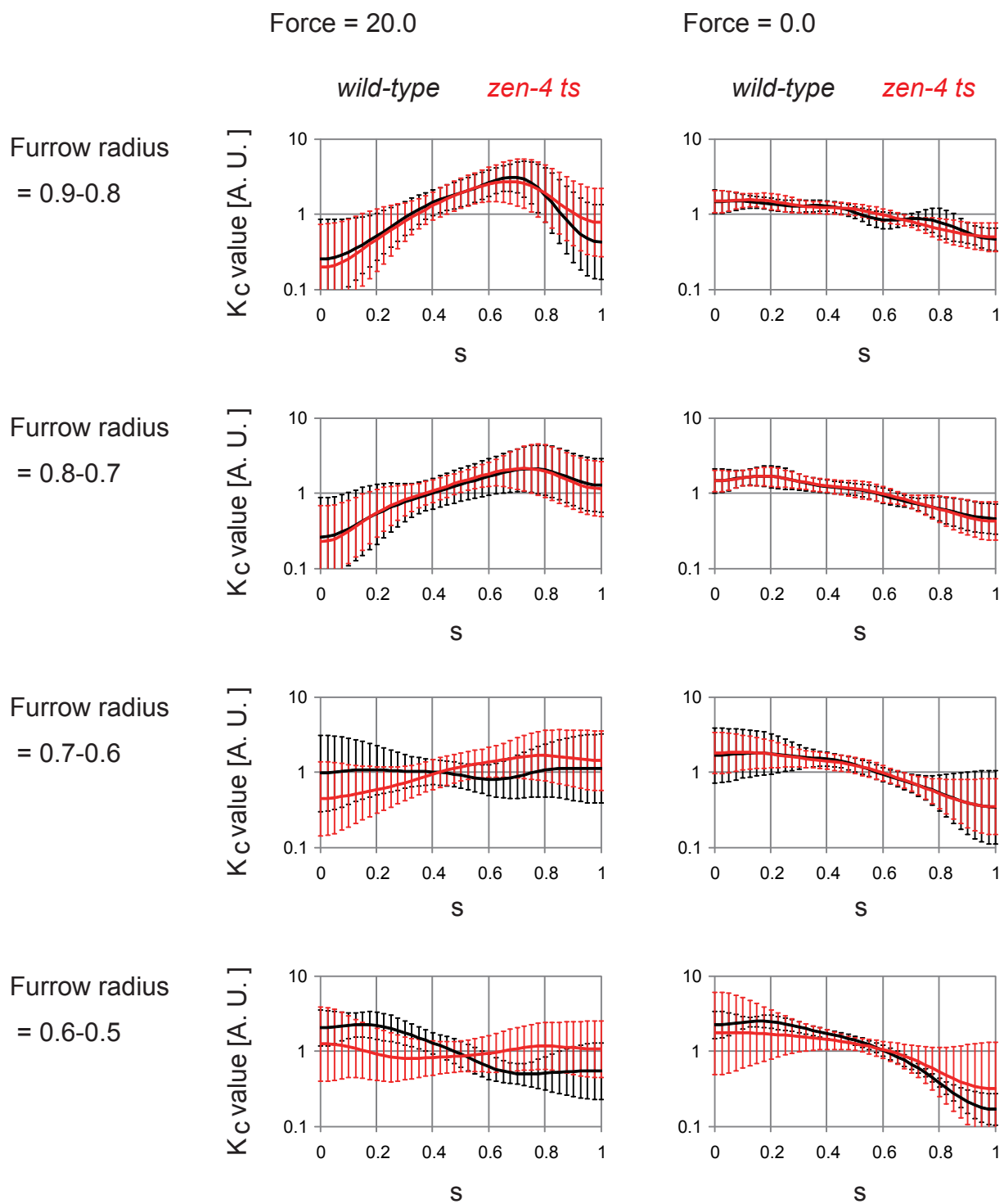

B

Force = 50.0

Furrow radius  
= 0.6-0.5

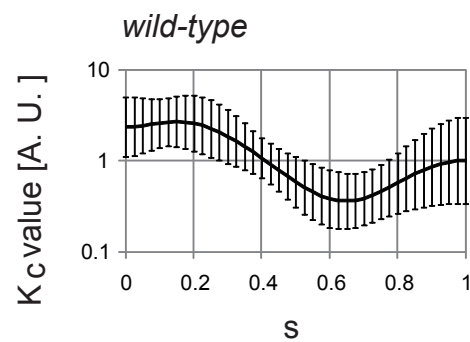

Figure S9

Supplement: Figure S9 — Comparison of the spatio-temporal changes in Kc between wild-type and zen-4 ts cells. (A) Kc estimated under Force = 20.0 or = 0.0 for the presented furrow radii. Black, wild-type cells; red, zen-4 ts cells. (B) Kc estimated under Force = 50.0 in the wild-type cells are shown. The Kc values were still spatially inconstant. (PDF) [file pone.0031607.s010.pdf]

A

Furrow radius = 0.6-0.5

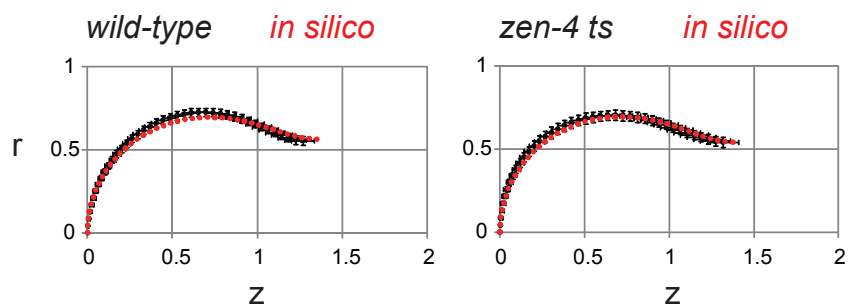

B

Furrow radius = 0.6-0.5

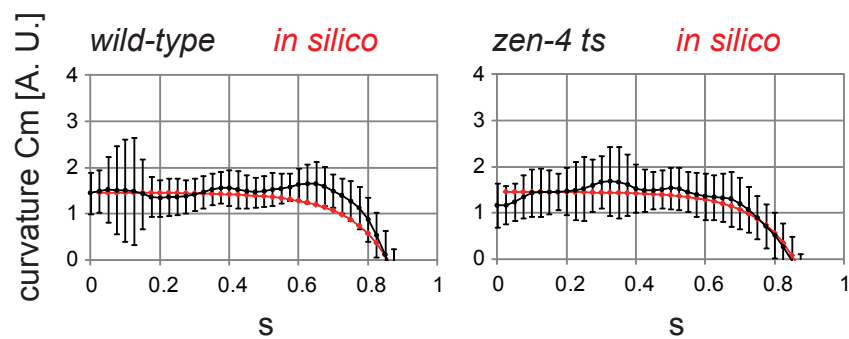

Figure S10

Supplement: Figure S10 — Comparison of the in vivo cell shapes in wild-type and zen-4 ts cells with those calculated under a spatially constant Kc . (A) Cell shapes were calculated in the bending model with a spatially constant Kc under Force = 20. The calculated shapes and the in vivo cell shapes in the wild-type and zen-4 ts cells were compared for a furrow radius = 0.6–0.5. N = 94 (wild-type) and 126 (zen-4 ts). (B) The curvatures Cm of the cell shapes in (A) are shown. The higher Cm around s = 0.6–0.8 observed in the wild-type cells disappeared in the zen-4 ts cells, and Cm in the zen-4 ts cells was consistent with that in the bending model under a spatially constant Kc. N = 94 (wild-type) and 126 (zen-4 ts). (PDF) [file pone.0031607.s011.pdf]

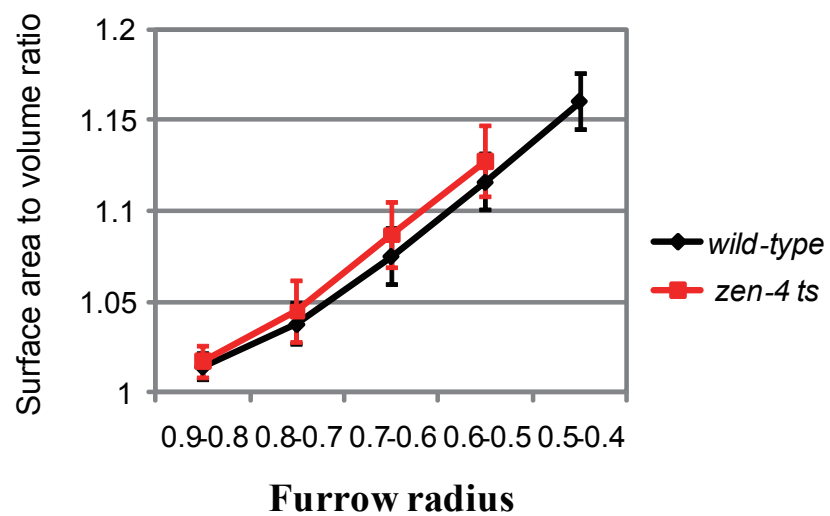

Figure S11

Supplement: Figure S11 — Comparison of the in vivo cell surface area in wild-type and zen-4 ts cells. The cell surface area normalized by cell volume was calculated for each furrow radius. The zen-4 ts cells had a slightly larger surface area than the wild-type cells. As a larger surface area is generally advantageous to form a deeper furrow, these results indicate that the status of the mechanical property, rather than the surface area, was more resistant to furrow ingression in the mutant cells than in the wild-type cells. N = 48, 70 (0.9–0.8), 96, 81 (0.8–0.7), 86, 96 (0.7–0.6), 94, 126 (0.6–0.5), and 98, n.e. (0.5–0.4) for each furrow radius (in parentheses) in the wild-type or zen-4 ts cells, respectively. (PDF) [file pone.0031607.s012.pdf]

A

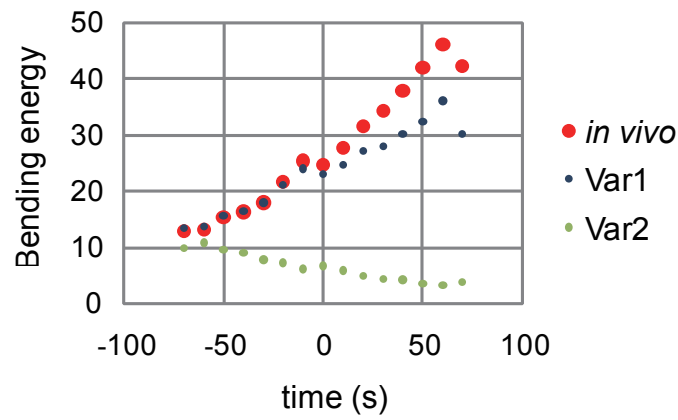

B

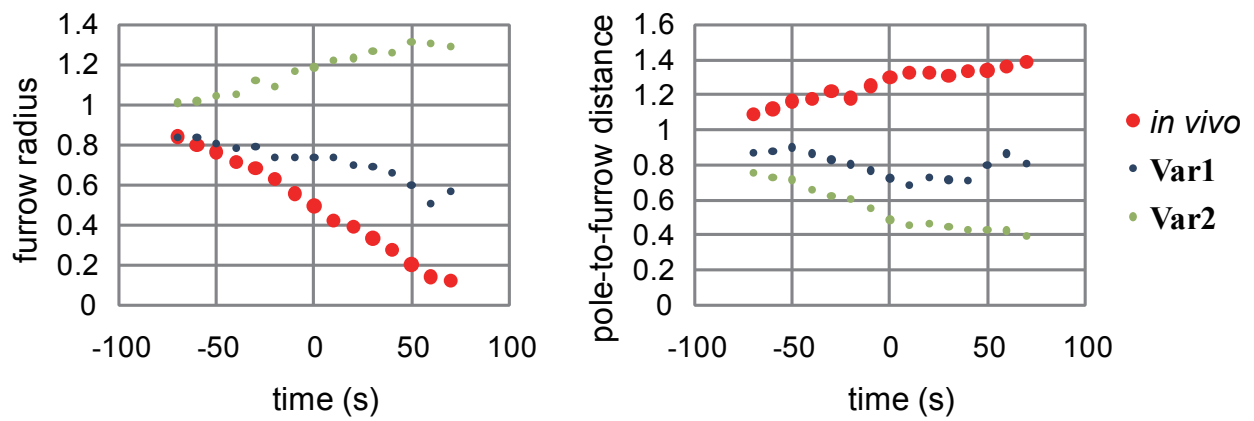

C

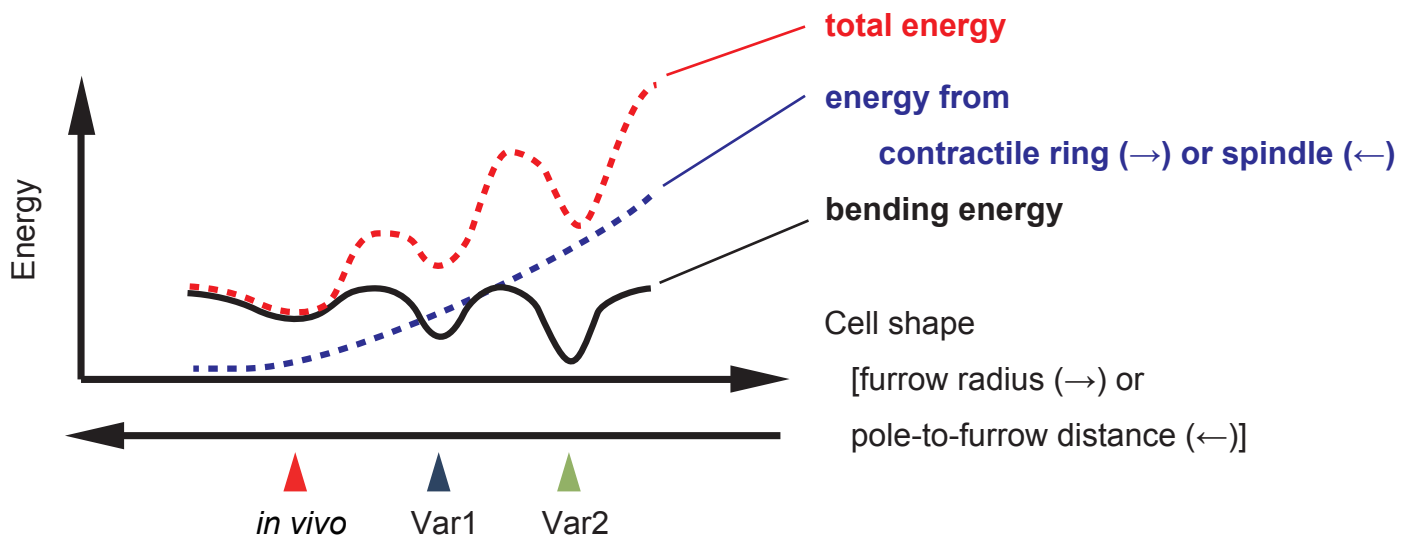

Figure S12

Supplement: Figure S12 — Comparison of the bending energy among various shapes with the same Kc values. (A) The values for the bending energy of various shapes presented in Figure 5B were calculated for each time point. Note that Figure 5B corresponds to a time of 40 s. These shapes had the same spatial distributions of Kc and were calculated under Force = 0, as described in Figure 5B. (B) The furrow radius and pole-to-furrow distance of each shape are shown. (C) Schematic illustration of the energy landscape and possible roles of bending elasticity, the contractile ring, and the spindle. An energy landscape at one time point was expected from (A) and (B). Note that “in vivo,” Var1, and Var2 were located at the global or local minimums of the bending energy (data not shown). Var1 and Var2 have larger furrow radii (→), smaller pole-to-furrow distances (←), and lower bending energy (black) than the in vivo shape. Therefore, additional energy sources (blue) may be required to stabilize the in vivo shape and avoid shape transformation from the in vivo shape to Var1 or Var2. One candidate was line tension energy derived from the contractile ring, which corresponds to 2πrγ in equation 4 in Section 4-1. However, the line tension energy was 0 in this analysis because contractile ring force (γ) was assumed to be absent. Nevertheless, it may be possible that the contractile ring can provide the energy source through another mechanism such as a kind of ratchet that confers a large amount of energy against furrow expansion, but no energy for furrow ingression. Spindle or cell adhesion, if it is present, may also provide an energy source. The sum of the energy landscape (black+blue) is shown in red, in which the in vivo shape is stabilized. Consistent with this ides, cell adhesion is critical for the progression of the contractile ring-independent cytokinesis in Dictyostelium discoideum and mammalian cultured cells [19], [27], [28]. The contractile ring-independent cytokinesis might be driven by the com [file pone.0031607.s013.pdf]

A

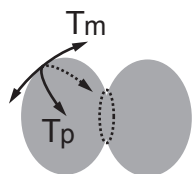

$$T_m \neq T_p$$

$T_m \neq \text{spatially const.}$

$T_p \neq \text{spatially const.}$

B

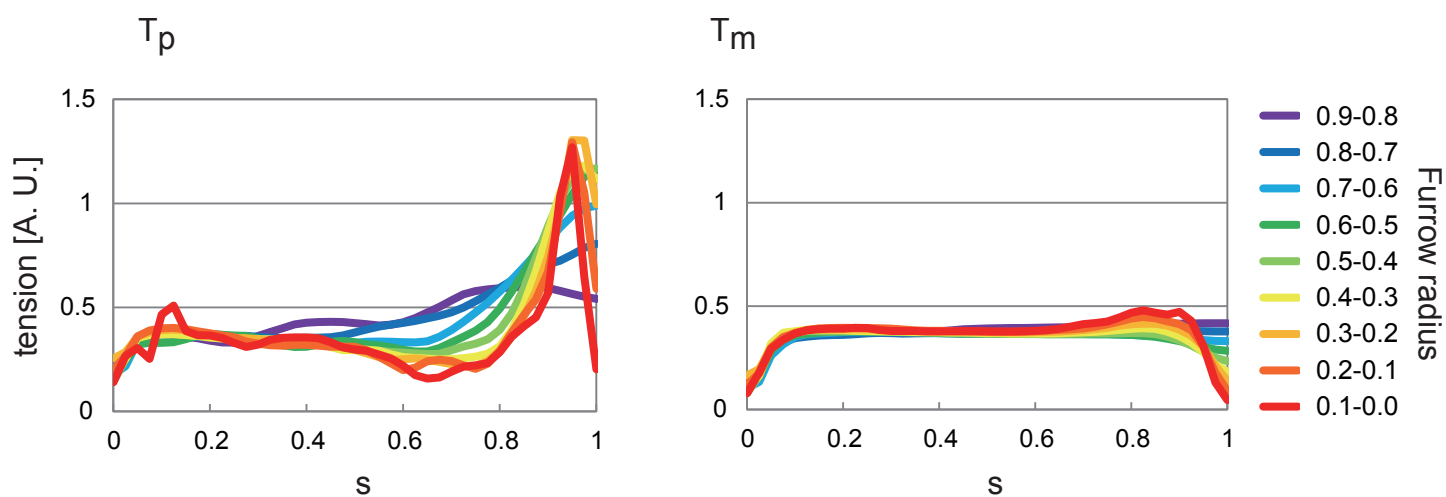

Figure S13

Supplement: Figure S13 — Estimation of the spatio-temporal changes in surface tension in the spatially inconstant surface tension model. (A) Schematic illustration of the surface tension model. (B) The estimated surface tension Tp and Tm are shown. The high Tp region around the furrow would correspond to the contractility of the contractile ring. (PDF) [file pone.0031607.s014.pdf]

Surface tension  $T_p$

Furrow radius  
= 0.9-0.8

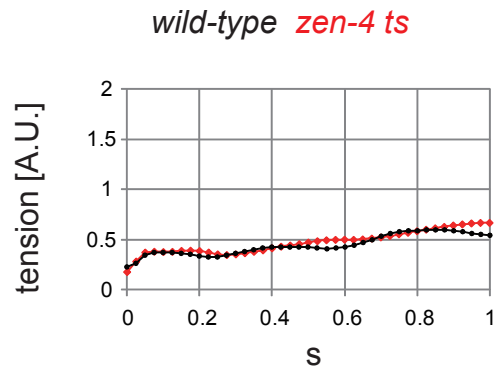

Furrow radius  
= 0.8-0.7

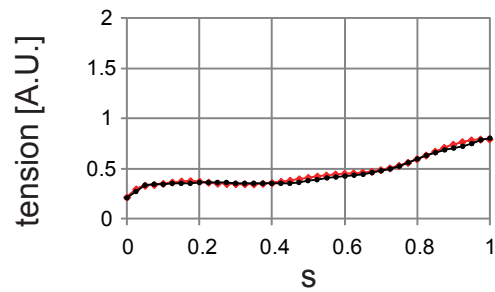

Furrow radius  
= 0.7-0.6

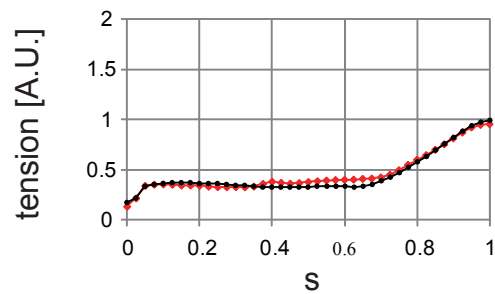

Furrow radius  
= 0.6-0.5

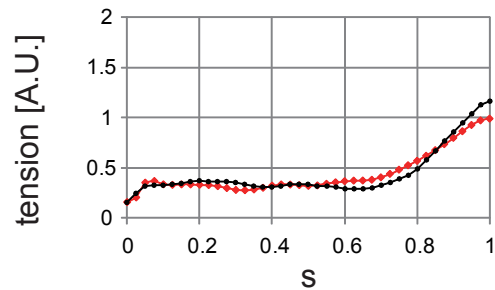

Figure S14

Supplement: Figure S14 — Comparison of the estimated spatio-temporal changes in surface tension Tp between wild-type and zen-4 ts cells. The values of the estimated surface tension Tp for each furrow radius are shown for wild-type and zen-4 ts cells. (PDF) [file pone.0031607.s015.pdf]
