# Supplementary material for: A High-Resolution Shape Fitting and Simulation Demonstrated Equatorial Cell Surface Softening during Cytokinesis and Its Promotive Role in Cytokinesis
Source: PLoS One. 2012 Feb 16;7(2):e31607. doi: 10.1371/journal.pone.0031607 (PMC3281004; doi:10.1371/journal.pone.0031607)
Supplement: Figure S3 — Quantified values of the r–z coordinates and curvatures. (A) The values of the r–z coordinate of the wild-type (black) and zen-4 ts (red) cells are shown for each furrow radius. The majority of the zen-4 ts cells arrested the furrow at a furrow radius of 0.6–0.5; thus, the values of the r–z coordinates in the zen-4 ts cells for a furrow radius <0.5 are not shown. N = 48, 70 (0.9–0.8), 96, 81 (0.8–0.7), 86, 96 (0.7–0.6), 94, 126 (0.6–0.5), 98, not shown (n.s.) (0.5–0.4), 76, n.s. (0.4–0.3), 62, n.s. (0.3–0.2), 53, n.s. (0.2–0.1), and 22, n.s. (0.1–0.0) for each furrow radius (in parentheses) in the wild-type or zen-4 ts cells, respectively. (B and C) The curvatures Cm (red) and Cp (blue) in the wild-type cells (B) or zen-4 ts cells (C) are shown for each furrow radius. The right panels are enlarged from the left ones. A region with a higher Cm in the wild-type cells is shown (B, red arrow heads). The larger error bars of Cp at s<0.2 may be caused by measurement errors, as described in Section 3 Quantification of cell shape. N for each furrow radius in the wild-type or zen-4 ts cells is shown in A. (D) Comparison of Cm between the wild-type and zen-4 ts cells. Cm in the wild-type cells from (B) and in the zen-4 ts cells from (C) are presented. (PDF) [file pone.0031607.s004.pdf]

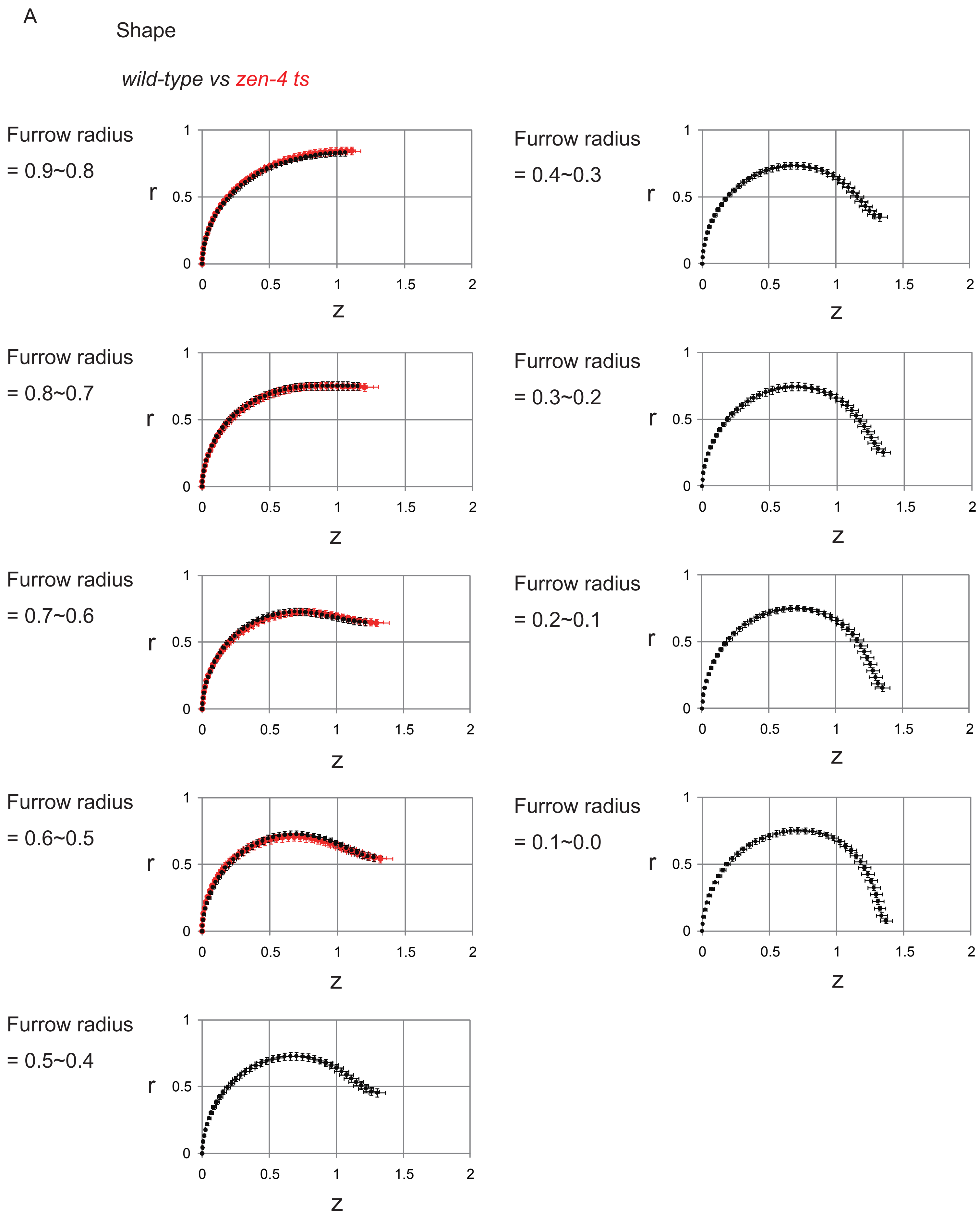
A
Shape
wild-type vs zen-4 ts
Furrow radius
= 0.9~0.8
Furrow radius
= 0.8~0.7
Furrow radius
= 0.7~0.6
Furrow radius
= 0.6~0.5
Furrow radius
= 0.5~0.4
Furrow radius
= 0.4~0.3
Furrow radius
= 0.3~0.2
Furrow radius
= 0.2~0.1
Furrow radius
= 0.1~0.0
r
z
0
0.5
1
1.5
2

Figure S3

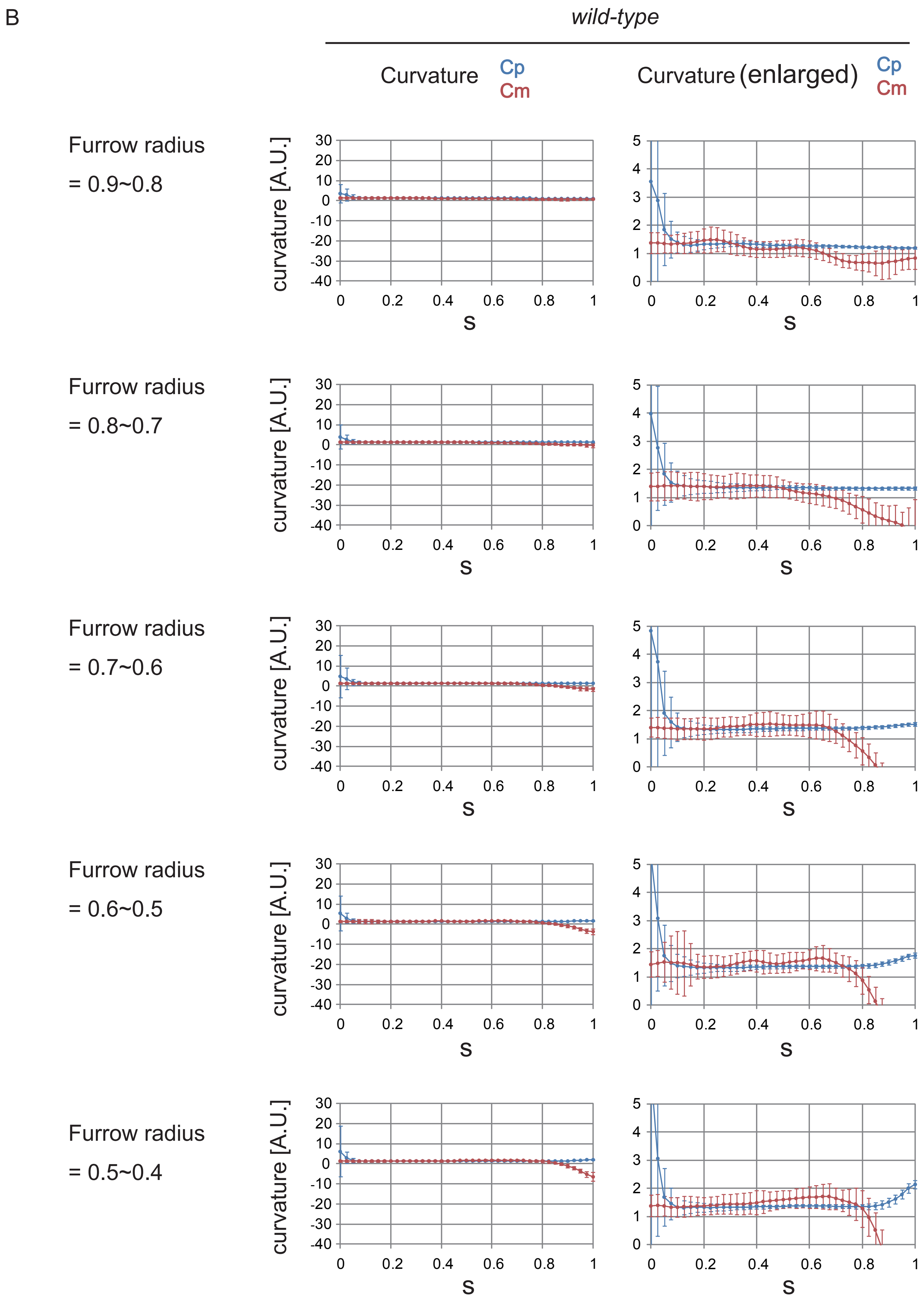

Figure S3 (continued)

B (continued)

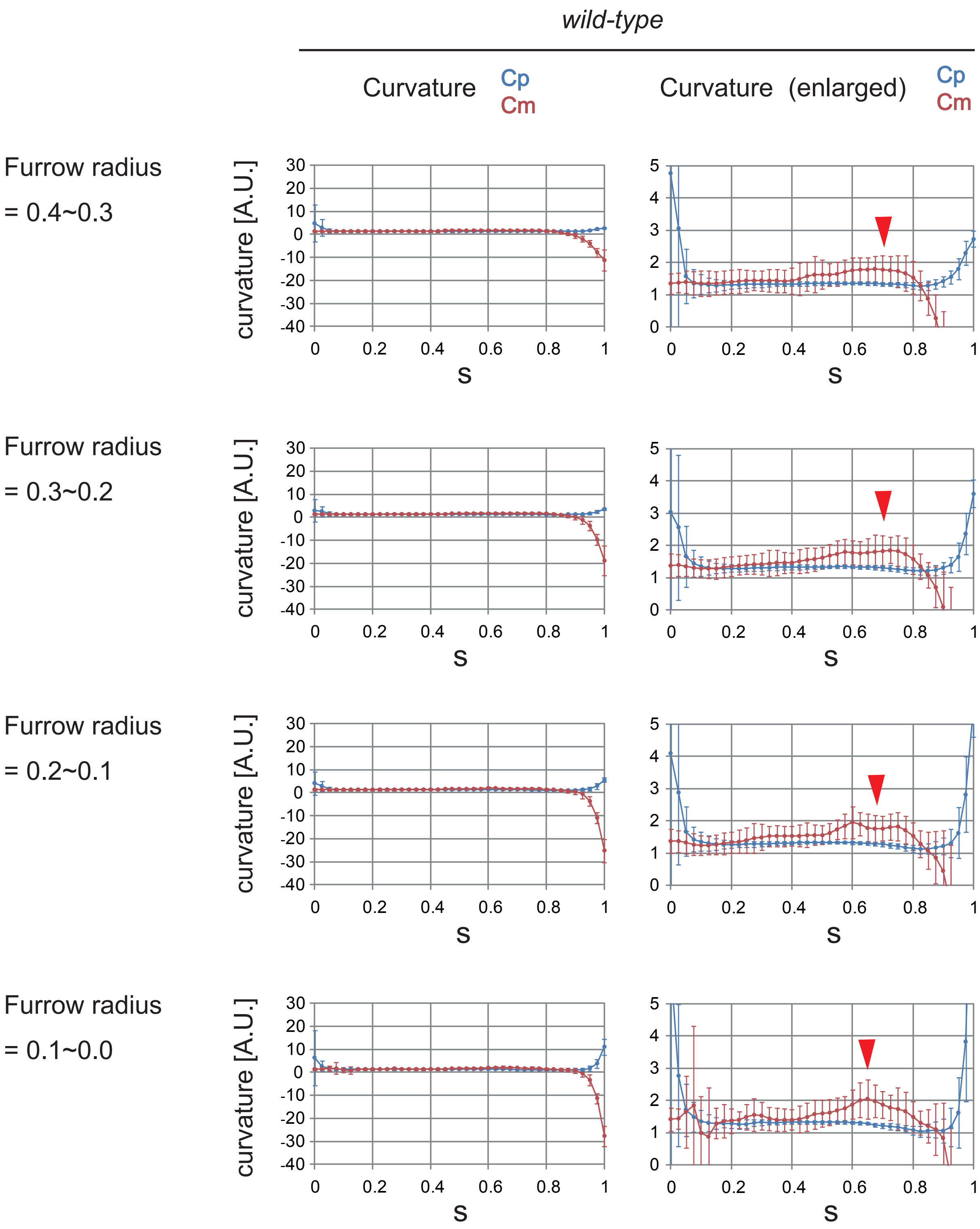

Figure S3 (continued)

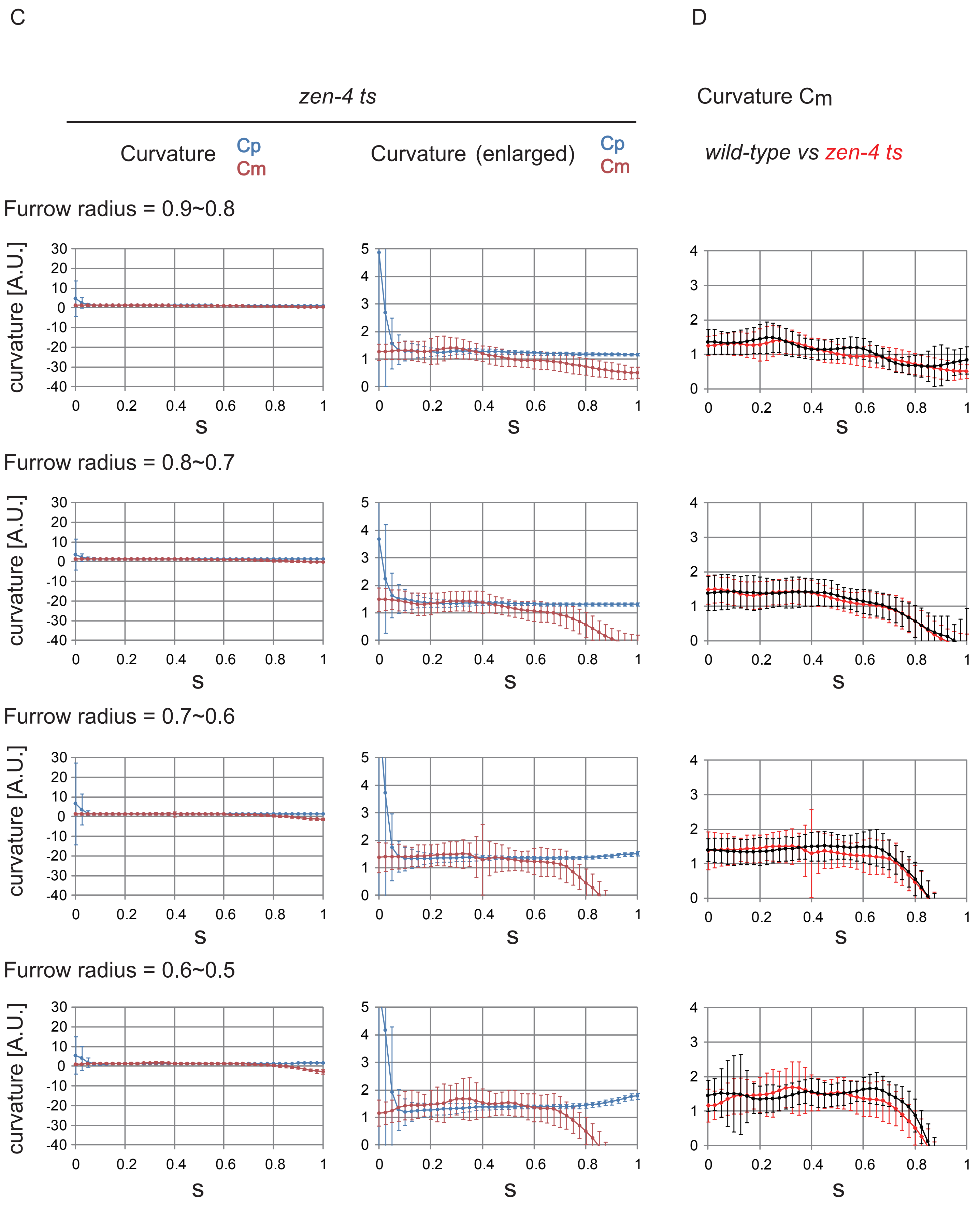

Figure S3 (continued)
